# Maternal γδ T Cells Shape Offspring Pulmonary Type-2 Immunity In A Microbiota-Dependent Manner

**DOI:** 10.1016/j.celrep.2023.112074

**Published:** 2023-02-13

**Authors:** Pedro H. Papotto, Bahtiyar Yilmaz, Gonçalo Pimenta, Sofia Mensurado, Carolina Cunha, Gina J. Fiala, Daniel Gomes da Costa, Natacha Gonçalves-Sousa, Brian H. K. Chan, Birte Blankenhaus, Rita G. Domingues, Tânia Carvalho, Matthew R. Hepworth, Andrew J. Macpherson, Judith E. Allen, Bruno Silva-Santos

**Affiliations:** 1 Instituto de Medicina Molecular João Lobo Antunes, Faculdade de Medicina, Universidade de Lisboa, Lisbon, Portugal; 2 Lydia Becker Institute for Immunology & Infection, Faculty of Biology, Medicine & Health, Manchester Academic Health Science Centre, University of Manchester, Manchester, United Kingdom; 3 Maurice Müller Laboratories, Department for Biomedical Research, University of Bern, Bern, Switzerland; 4 Department of Visceral Surgery and Medicine, Bern University Hospital, University of Bern, Bern, Switzerland; 5 Wellcome Centre for Cell-Matrix Research, Faculty of Biology, Medicine & Health, Manchester Academic Health Science Centre, University of Manchester, Manchester, United Kingdom

## Abstract

Immune development is profoundly influenced by vertically transferred cues. However, little is known about how maternal innate-like lymphocytes regulate offspring immunity. Here we show that mice born from γδ T cell-deficient (TCRδ^-/-^) dams display an increase in first-breath-induced inflammation, with a pulmonary milieu selectively enriched in type-2 cytokines, and type-2-polarized immune cells, when compared to the progeny of γδ T cell-sufficient dams. Upon helminth infection, mice born from TCRδ^-/-^ dams sustain an increased type-2 inflammatory response. Surprisingly, this was independent of the genotype of the pups. Instead, the offspring of TCRδ^-/-^ dams harbors a distinct intestinal microbiota, acquired during birth and fostering, and decreased levels of intestinal short-chain fatty acids (SCFA), such as pentanoate and hexanoate. Importantly, exogenous SCFA supplementation inhibits type-2 innate lymphoid cell function and suppresses first-breath- and infection-induced inflammation. Taken together, our findings unravel a maternal γδ T cell – microbiota – SCFA axis regulating neonatal lung immunity.

## Introduction

The development of the immune system is particularly sensitive to maternally derived factors^1^. Maternal transfer of bacterial species during pregnancy and pre-weaning is responsible for determining the offspring’s microbiota profile, which in turn influences immune responses later in life^2–4^. For example, vertical transfer of bacterial products via antibodies during pregnancy was shown to modulate the maturation of the intestinal immune system in the offspring^5^. Maternally transferred antibodies were also recently shown to control the homeostatic number of colonic regulatory T (T_reg_) cells across generations^6^. Furthermore, maternal exposure to a high fiber diet during pregnancy leads to decreased airway allergic disease severity in their progeny, through the transfer of short-chain fatty acids (SCFA), a major class of dietary metabolites^7^. Interestingly, airway immune disorders seem to be tightly connected with early life exposure to environmental challenges^8^ likely due to type-2 immune activation and tissue remodeling characterizing the perinatal phase of lung development^9,10^. Apart from exciting biological consequences, this maternal crosstalk has profound implications on the design of experiments using genetically engineered mice, namely the choice of controls to generate accurate and reproducible data. Even though littermate controls are considered the gold standard^11^, it is clear that alternative approaches, such as co-housing and especially genetic background matching (to so-called “wild type” mice, often obtained directly from commercial vendors), are still widely used in biomedical research. However, given that many external cues are vertically transferred during early life and have long-lasting consequences^5–7,12^, controlling for environmental factors without using littermate controls is nearly impossible. On a positive note, the problems evidenced by the use of suboptimal controls raise interesting questions on how parental-derived factors affect the offspring^13,14^. In particular, even though the maternal influence on progeny immune system is evident, the role of individual components of the maternal immune system (beyond antibodies) in regulating the immune status of the offspring remains largely unexplored.

γδ T cells are a population of innate-like lymphocytes known to reside in different tissues that compose the maternal-newborn interface, such as the female reproductive tract^15–17^, the skin^18,19^ and mammary gland^20^. Murine γδ T cells were shown to control antibody production both under steady state conditions^21^ and upon challenge^22^. Also, γδ T cells are known to participate in an intricate crosstalk with barrier tissue microbiota^23,24^, notably being able to exert significant selective pressure on bacterial communities, as recently documented for the oral microbiome^25,26^. Hence, we aimed to determine if and how maternal γδ T cells affect offspring immune system development. Here we show that pups born from TCRδ^-/-^ (compared to TCRδ^+/-^) dams present a perinatal pulmonary environment strongly biased towards type-2 immunity, both in the steady state and upon helminth infection, irrespective of their own genotype. Based on cross-fostering and cesarean section (C-Sec) delivery experiments, as well as antibiotic treatment and metabolomic approaches, we propose a critical role for the microbiota and their metabolic byproducts in this process.

## Results

### Absence of maternal γδ T cells exacerbates perinatal pulmonary type-2 immune activation in offspring

**Figure 1:**
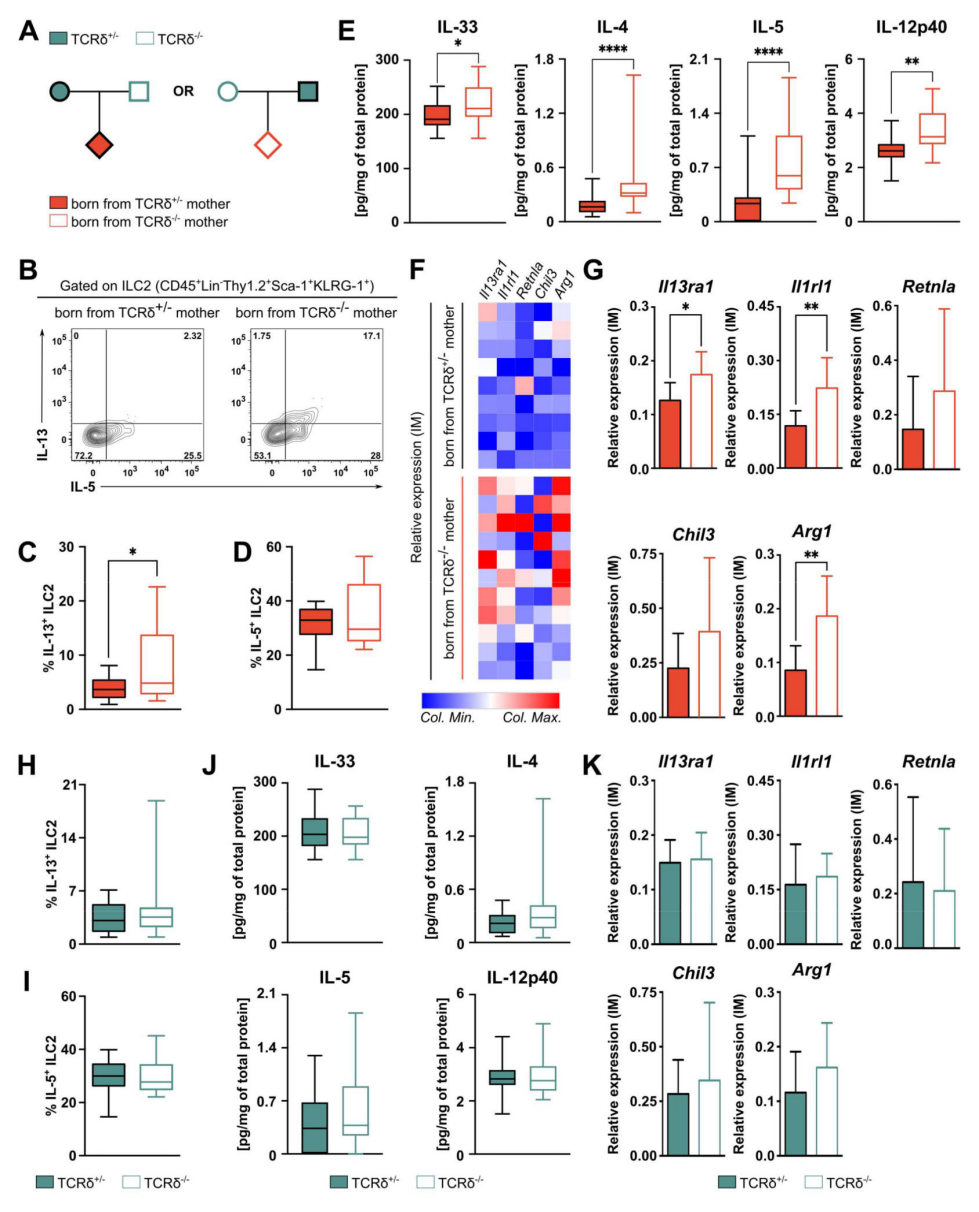
Absence of maternal γδ T cells exacerbates first-breath-induced type-2 inflammation in the offspring. **(A)** Breeding strategy employed to evaluate the gene-extrinsic and gene-intrinsic roles of γδ T cell depletion on the formation of neonatal pulmonary immune system. **(B)** Flow cytometry analysis of intracellular IL-5 and IL-13 in ILC2s isolated from the lungs of pups described in (A) and stimulated *in vitro* for 3h in the presence of PMA, Ionomycin and Brefeldin A. Frequencies of **(C)** IL-13^+^ and **(D)** IL-5^+^ cells within the ILC2 population in the lungs **(E)** Concentration of cytokines within the whole lung homogenate normalized by total protein **(F)** Heatmap and **(G)** bar plots depicting mRNA expression (relative to *Hprt* and *Actb*) of selected genes by qPCR in sorted interstitial macrophages (IM) from the pups described in (A), grouped by maternal genotype. Frequencies of **(H)** IL-13^+^ and **(I)** IL-5^+^ cells within the ILC2 population in the lungs of pups depicted in (A), grouped by offspring genotype. **(J)** Concentration of cytokines within the whole lung homogenate normalized by total protein **(K)** mRNA expression of selected genes by qPCR in sorted IM, grouped by offspring genotype. Data pooled from at least three independent litters per genotype **(B-D; H-I)** n= 16-17 mice per group. **(E-J)** n= 18-23 mice per group. **(G, K)** n= 9-11 mice per group **(C-E; H-J)** Box-and-whisker plots display first and third quartiles, and the median; whiskers are from each quartile to the minimum or maximum. **(G, K)** Error bars represent Mean ±SD. Normality of the samples was assessed with D’Agostino Pearson normality test; statistical analysis was then performed using Student’s *t* test or Mann-Whitney test. * *p* < 0.05; ** *p* < 0.01; **** *p* < 0.0001

The importance of pulmonary γδ T cells in the establishment of lung type-2 immune responses has been highlighted by various groups^27–29^. While trying to understand the mechanisms underlying the γδ T cell – type-2 immunity crosstalk, we observed that mice from our TCRδ^-/-^ colony (TCRδ^-/-^ x TCRδ^-/-^) possessed decreased frequencies and numbers of pulmonary ILC2, when compared to age- and sex-matched adult C57BL6/J mice (Figures S1A and S1B). However, and intriguingly, when we compared TCRδ^+/+^ and TCRδ^-/-^ littermates from heterozygous breedings (TCRδ^+/-^ x TCRδ^+/-^), no differences were observed in lung ILC2, suggesting this was not dependent on offspring genotype (Figure S1C and S1D). Moreover, when infected with the helminth *Nippostrongylus brasiliensis*, a parasite that induces a damage/repair cycle in the lungs that is highly dependent on type-2 immunity^30^, TCRδ^-/-^ mice presented increased lung damage compared to C57BL6/J mice (Figures S1E and S1F). However, TCRδ^-/-^ and TCRδ^+/+^ littermates showed comparable lung damage upon infection (Figures S1G and S1H). As our TCRδ^+/-^ breeding is derived from the TCRδ^-/-^ colony and both experience the same environmental factors, we hypothesized that absence of γδ T cells in the dams could be responsible for altered lung ILC2. To test this hypothesis, we established reciprocal breeding colonies by crossing TCRδ^-/-^ females with TCRδ^+/-^ males and vice-versa; these breeding strategies give rise to both TCRδ^-/-^ and TCRδ^+/-^ progeny allowing us to exclude gene-intrinsic effects in the offspring (Figure 1A). We chose to analyze the lungs of pups born from γδ-deficient and -sufficient dams around post-natal day (PN) 15, as the main events leading to the maturation of the pulmonary immune system peak during this time as a consequence of the first-breath-induced inflammatory reaction^9,10^. In accordance with our hypothesis, pups born from TCRδ^-/-^ dams exhibited a decrease in lung ILC2, when compared to pups born from TCRδ^+/-^ dams (Figures S1J and S1K). Interestingly, the offspring of TCRδ^-/-^ dams displayed augmented perinatal pulmonary type-2 activation, including increases in IL-13^+^, but not IL-5^+^, ILC2 (Figures 1B–D), and IL-5^+^ mast cells (Figures S1N–P). Accordingly, we also observed increased tissue levels of the type-2-inducing alarmin IL-33, and the type-2 cytokines IL-4 and IL-5, in the lungs of the progeny of TCRδ^-/-^ dams, when compared with pups born from their TCRδ^+/-^ counterparts (Figure 1E). In addition, consistent with findings in type-2 asthma models^31^, IL-12p40, but not IL-12p70 and IL-23, was also increased in the lungs of the offspring from TCRδ^-/-^ dams (Figures 1E and S1S). Moreover, interstitial macrophages from the offspring of TCRδ^-/-^ dams exhibited a significant, albeit small, increase in the expression of type-2-regulated genes, such as *Il13ra, I1rl1*, and *Arg1* (Figure 1F and 1G). Critically, when the same pups were stratified according to their own genotype, no differences were observed between TCRδ^-/-^ and TCRδ^+/-^ pups regarding lung ILC2 (Figures 1H, 1I, S1L and S1M), mast cells (Figures S1Q and S1R), lung cytokine levels (Figure 1J), or gene expression on interstitial macrophages (Figure 1K). Thus, the observed increase in first-breath-induced type-2 immune responses in pups born from TCRδ^-/-^ dams depends solely on the maternal genotype.

Of note, despite the differences observed in pulmonary type-2 immunity, pups born from TCRδ^-/-^ and TCRδ^+/-^ dams presented a similar distribution of lung myeloid populations (Figure S2A), T and B lymphocytes (Figure S2B), and T_reg_ cells (Figure S2C). Moreover, the expression of epithelial genes controlling surfactant and mucin production, and barrier integrity was similar between pups from both breeding strategies (Figure S2D). Finally, the differences between the progeny of TCRδ^-/-^ and TCRδ^+/-^ dams seemed restricted to the lung, as we found similar frequencies of ILC2 precursors and other lymphoid and myeloid progenitors in the bone marrow (Figure S3A), and of innate lymphoid cells in the small intestine lamina propria (Figure S3B). These data indicate that maternal γδ T cells “vertically” (and selectively) regulate first-breath-induced pulmonary reaction in the offspring.

### Microbiota transfer in early life mediates differences in the pulmonary immune system of the offspring from TCRδ^+/-^ and TCRδ^-/-^ dams

**Figure 2:**
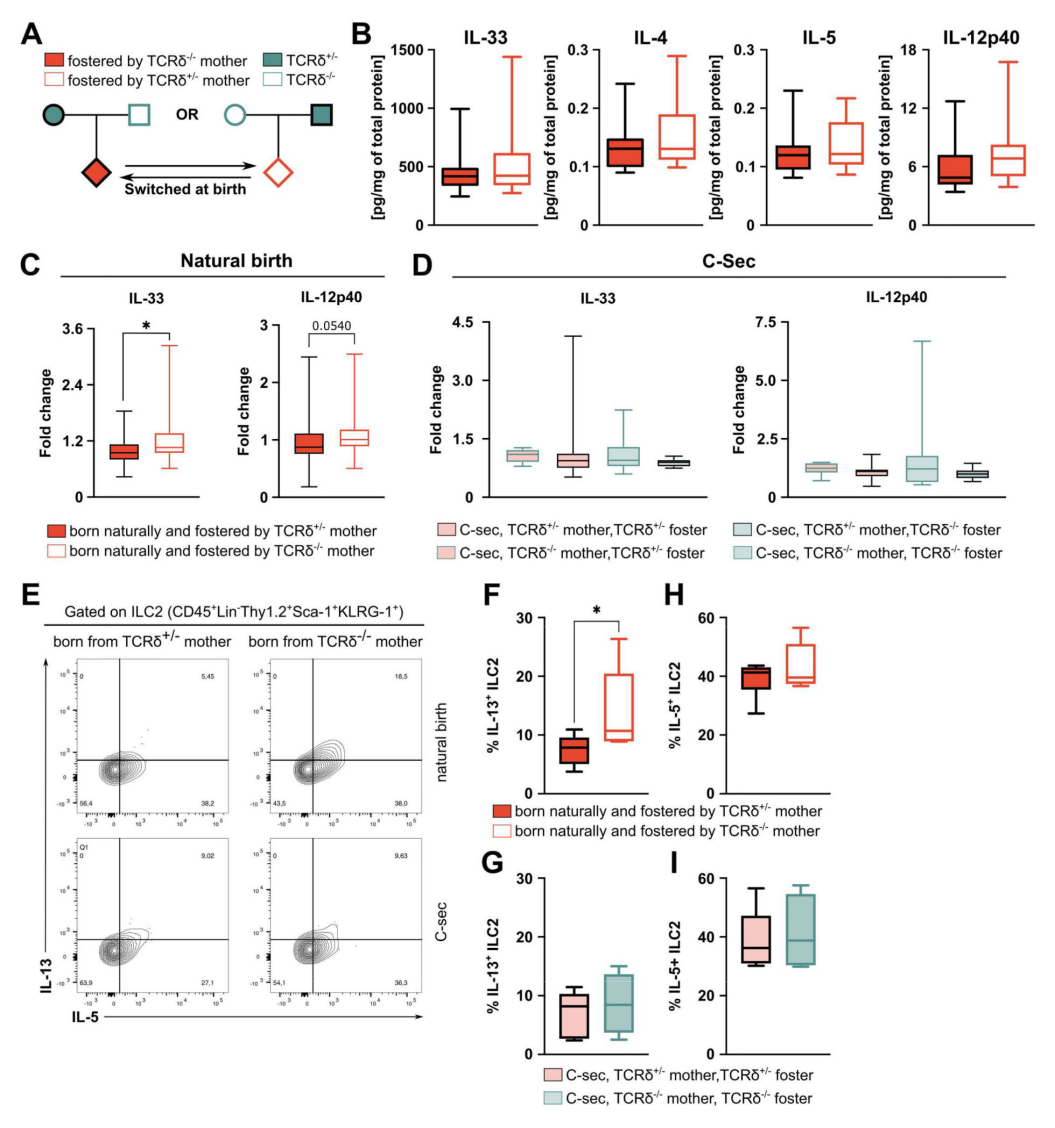
Maternal regulation of first-breath-induced inflammation in the offspring occurs during delivery and fostering. **(A)** Breeding strategy employed to evaluate the role of fostering on perinatal lung inflammation. **(B)** Concentration of cytokines within the whole lung homogenate; concentration was normalized to the total protein in the tissue. **(C-D)** Concentration of cytokines within the whole lung homogenate of pups born naturally or via C-Sec from TCRδ^+/-^ or TCRδ^-/-^ dams grouped by maternal and foster genotype; concentration was normalized to the fold change relative to pups born naturally from TCRδ^+/-^ dams. **(E)** Flow cytometry analysis of intracellular IL-5 and IL-13 in ILC2s isolated from the lungs and stimulated *in vitro* for 3h in the presence of PMA, Ionomycin and Brefeldin A. Frequencies of **(F-G)** IL-13^+^ and **(H-I)** IL-5^+^ cells within the ILC2 population in the lungs, grouped by maternal genotype and mode of delivery. **(B)** Data pooled from three independent litter swaps per maternal genotype; n= 18-21 mice per group. **(C-D)** Data pooled from three independent litters per maternal genotype; n= 35-7 mice per group. **(E-G)** Data from one litter per maternal genotype; n= 4-6 mice per group. **(B; D; F-G)** Box-and-whisker plots display first and third quartiles, and the median; whiskers are from each quartile to the minimum or maximum. Normality of the samples was assessed with D’Agostino Pearson normality test; statistical analysis was then performed using Student’s *t* test or Mann-Whitney test. * *p* < 0.05

Transfer of maternally-derived factors during pregnancy has been shown to regulate the development of the intestinal immune system^5,6^ and suppress airway inflammation in the offspring ^7^. Surprisingly, cross-fostering pups born from TCRδ^-/-^ dams with TCRδ^+/-^ surrogates, and vice-versa (Figures 2A), abrogated the differences observed in the pulmonary milieu between their respective progeny (Figure 2B, compared to Figure 1E). This suggested that the vertical conditioning of offspring lung immunity did not take place exclusively during pregnancy. To further rule out a role for maternally transferred factors during gestation and delivery, pups born via C-Sec from either TCRδ^-/-^ or TCRδ^+/-^ dams were co-fostered with pups born naturally (NB) by either a mother of the same or the opposite genotype (Figures 2C and 2D). Consistently, whereas NB pups from TCRδ^-/-^ dams retained increased lung levels of IL-33, and IL-13-producing pulmonary ILC2, C-Sec delivery disrupted the increase in perinatal type-2 activation in the lungs of pups from TCRδ^-/-^ dams, irrespective of the foster mother (Figures 2D–I). Of note, fostering pups born via C-Sec from TCRδ^+/-^ dams on their TCRδ^-/-^ counterparts did not induce an increase in lung inflammation (Figure 2D).

**Figure 3:**
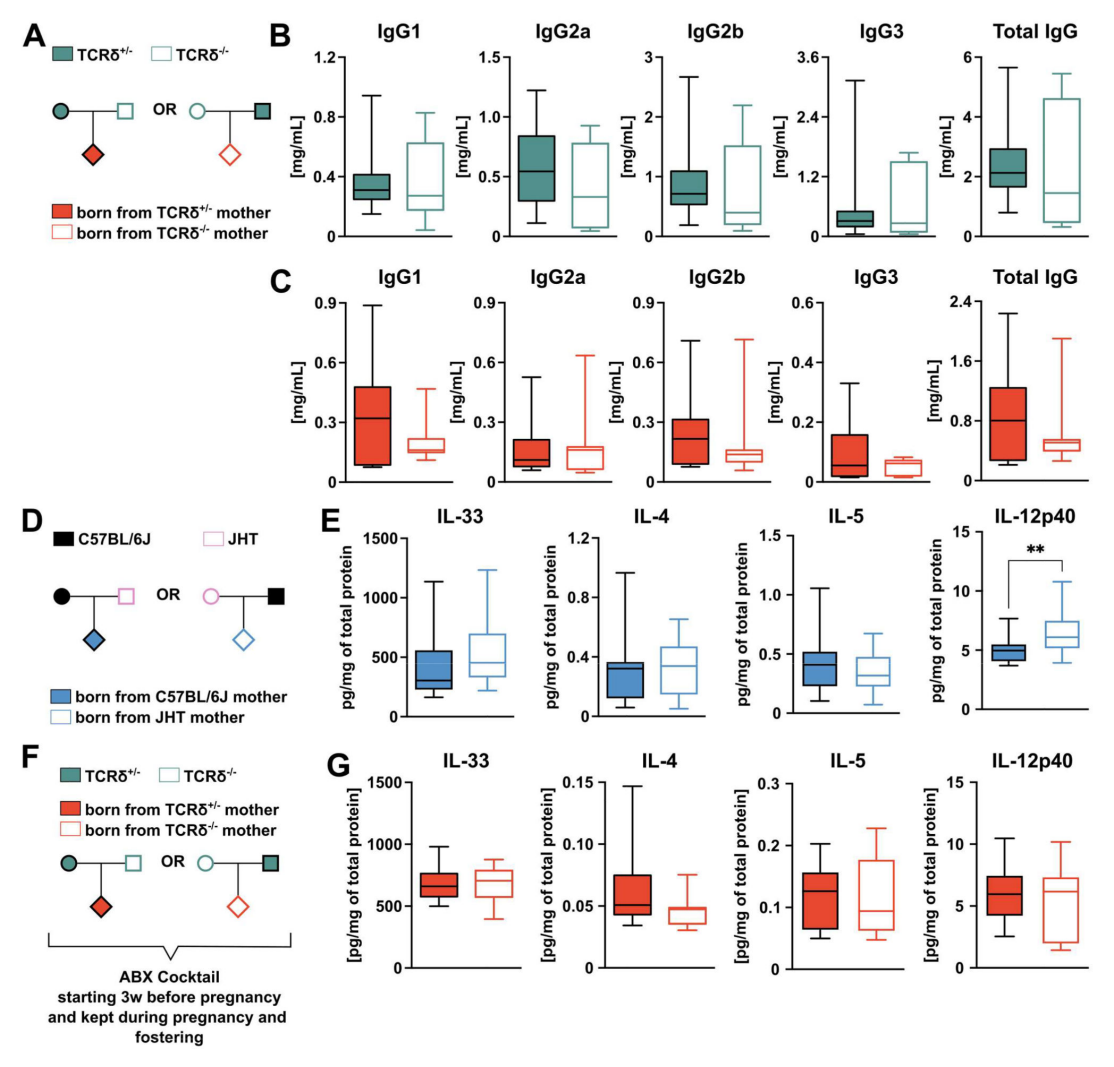
Maternal regulation of first-breath-induced inflammation in the offspring is microbiota-dependent but antibody-independent. **(A)** Breeding strategy. Concentration of total IgG and its subclasses in the serum of **(B)** TCRδ^+/-^ and TCRδ^-/-^ dams and their **(C)** progeny. **(D)** Breeding strategy employed to evaluate if maternal transfer of antibodies impact on perinatal lung inflammation. **(E)** Concentration of cytokines within the whole lung homogenate normalized by total protein, grouped by maternal genotype. (**F)** Breeding strategy employed to evaluate the role of microbiota in mediating maternal γδ T cell conditioning of the pulmonary immune system of the offspring. **(G)** Concentration of cytokines within the whole lung homogenate normalized by total protein, grouped by maternal genotype. **(B)** n= 7-14 mice per group. **(C)** n= 19-21 mice per group. **(E)** n= 18-22 mice per group. **(G)** n= 17-18 mice per group. **(C-G)** Data pooled from three independent litters per maternal genotype **(B-C; E; G)** Box-and-whisker plots display first and third quartiles, and the median; whiskers are from each quartile to the minimum or maximum. Normality of the samples was assessed with D’Agostino Pearson normality test; statistical analysis was then performed using Student’s *t* test or Mann-Whitney test. ** *p* < 0.01

Maternal antibodies have been shown to prevent the development of asthma in the offspring through the control of aberrant type-2 immune responses^32^, and γδ T cells can regulate natural and adaptive antibody production^21,22^. However, the circulating levels of total IgG and IgG subclasses were similar between both TCRδ^-/-^ and TCRδ^+/-^ dams (Figures 3A and 3B) and their respective progeny (Figures 3A and 3C). Futhermore, we established reciprocal breeding colonies by crossing antibody-deficient JHT females with antibody-sufficient males, and vice-versa (Figure 3D). As expected, the offspring of both JHT dams and their WT counterparts presented similar tissue levels of type-2 cytokines IL-33, IL-4, and IL-5 in their lungs, and a slight increase in IL-12p40 (Figure 3E).

Vertical transfer of microbiota^4^ or its products^5^ have also been implicated in modulating offspring immunity. Hence, we treated parents and progeny from our reciprocal breeding strategies with a wide spectrum antibiotics (ABX) cocktail, starting from before pregnancy through gestation and fostering (Figure 3F). In contrast with the control setting (Figure 1E), pups born from ABX-treated TCRδ^-/-^ and TCRδ^+/-^ dams presented similar tissue levels of cytokines in their lungs (Figure 3G), suggesting that the enhanced type 2 pulmonary responses observed in pups born to TCRδ^-/-^ dams resulted from vertical transfer of microbiota or microbial-derived molecules.

### TCRδ^-/-^ dams co-housed with TCRδ^+/-^ males retain differences in cutaneous bacterial communities

**Figure 4:**
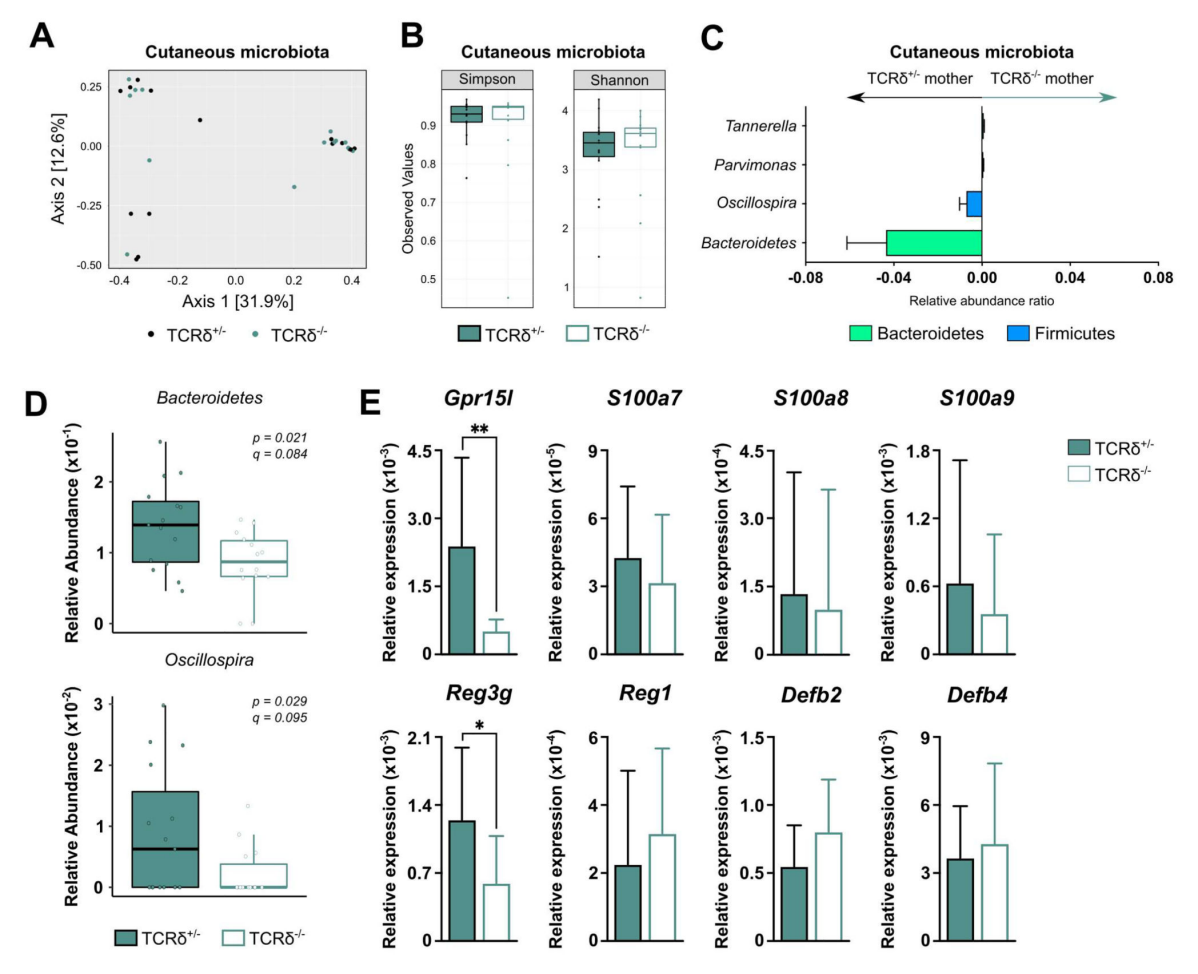
TCRδ^+/-^ and TCRδ^-/-^ dams display discrete skin microbiota and antimicrobial peptide profiles even after co-housing. Microbial composition differences in **(A-B)** skin microbiota from TCRδ^+/-^ and TCRδ^-/-^ dams are plotted using **(A)** beta diversity (Bray–Curtis dissimilarity PCoA) and **(B)** alpha diversity (Shannon and Simpson indices). **(C)** Skin microbiota taxa associated with TCRδ^+/-^ and TCRδ^-/-^ dams and plotted as relative abundance ratios. **(D)** Relative abundance of taxa significantly enriched (p < 0.05, q < 0.2) in the skin microbiota of TCRδ^+/-^ dams. **(E)** Gene expression of AMP genes in total skin tissue from TCRδ^+/-^ and TCRδ^-/-^ dams normalized to the average expression of *18S and Actb*. **(A-D)** n= 14-15 mice per group. **(E)** n= 10 mice per group. Box-and-whisker plots display first and third quartiles, and whiskers are from each quartile to the minimum or maximum. Normality of the samples was assessed with D’Agostino Pearson normality test; statistical analysis was then performed using Student’s *t* test or Mann-Whitney test. * *p* < 0.05; ** *p* < 0.01.

Co-housing is known to highly homogenizes the microbiota between different mouse colonies^33^. Thus, for our breeding setup, we expected to observe no differences in the microbiota of TCRδ^-/-^ and TCRδ^+/-^ dams upon co-housing with a male from the opposing genotype. In fact, before establishment of the reciprocal breeding pairs, TCRδ^-/-^ and TCRδ^+/-^ females displayed significant differences in fecal bacterial diversity and richness (Figures S4A and S4B). As expected, after breeding establishment, beta diversity analysis of the maternal fecal microbiota showed a significant change in bacterial diversity of TCRδ^-/-^ and TCRδ^+/-^ females before and after co-housing (Figures S4C and S4D); no changes in bacterial richness were observed (Figures S4E and S4F). As a result, after the establishment of breeding pairs, TCRδ^-/-^ and TCRδ^+/-^ dams displayed similar bacterial diversity and richness in their fecal (Figures S5A and S5B), vaginal (Figures S5C and S5D) and cutaneous (Figures 4A and 4B) microbiota. However, comparison of the relative abundances of specific skin microbial communities showed a significant increase of Bacteroidetes phyla and *Oscillospira* genus in TCRδ^+/-^ dams (Figures 4C and 4D), and a minor increase of *Parvimonas* and *Tannerella* genera in TCRδ^-/-^ dams (Figure 4C). Hence, even though the co-housing process largely homogenizes the microbiota, taxa-specific differences in the skin microbiota are retained between TCRδ^+/-^ and TCRδ^-/-^ dams. Given that γδ T cells often act through the secretion of cytokines, and are major producers of IL-17A and IL-22^34^, both of which have been implicated in the regulation of commensal microbes^35,36^, we employed the reciprocal breeding strategy using *Il17a*^-/-^ and *Il22*^-/-^ mice in order to investigate if these cytokines were implicated in the regulation of neonatal pulmonary immune responses (Figures S6A and S6C). Surprisingly, and in clear contrast to what we observed in the progeny of the TCRδ^-/-^ dams (Figure 1E), the offspring of *Il17a*^-/-^ and *Il22*^-/-^ dams exhibited a decrease in the levels of lung IL-33 (Figures S6B and S6D), IL-5 (Figure S6B), and IL-12p40 (Figure S6D) when compared to the offspring of control dams. As an alternative mechanism, we next evaluated the expression of multiple antimicrobial peptides (AMP; Ahuja et al., 2017; Kumar et al., 2016) in the skin of TCRδ^+/-^ and TCRδ^-/-^ dams. Interestingly, even though we observed no differences in the expression of the genes encoding S100 proteins (*S100a7, S100a8* and *S100a9*), defensins (*Defb2* and *Defb4*) or Reg1 (*Reg1*), TCRδ^-/-^ dams showed significantly lower expression of *Reg3g* and *Gpr15l*, encoding the AMP Reg3-γ and GPR-15L, respectively (Figure 4E). Of note, we found no differences in the expression of AMP genes in vaginal tissue (Figure S7A). Thus, the regulation of particular AMPs, such as Reg3-γ and GPR-15L, selectively in the skin, may explain how γδ T cells shape local commensal bacterial communities.

### The progeny of TCRδ^+/-^ dams acquires postnatally an intestinal microbiota with increased capacity to produce SCFA

**Figure 5:**
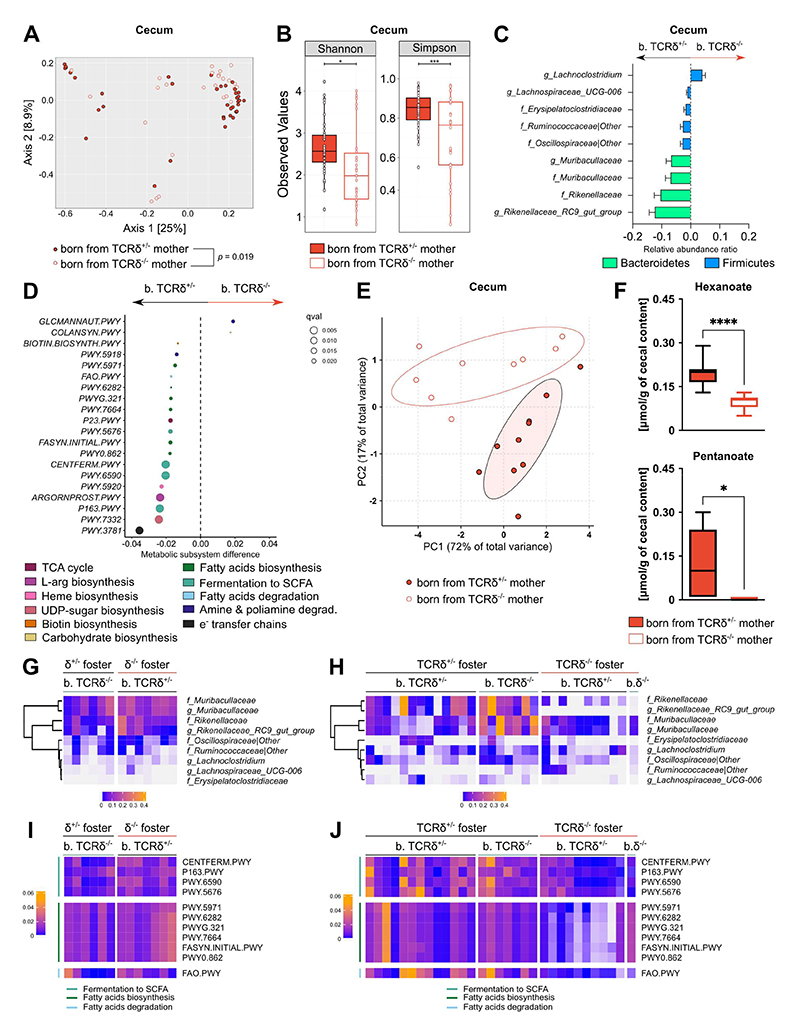
The offspring of γδ-sufficient dams acquire postnatally a discrete gut microbiota with increased SCFA production compared to pups born from γδ-deficient dams. Microbial composition differences in cecum microbiota from pups born from TCRδ^+/-^ and TCRδ^-/-^ dams are plotted using **(A)** beta diversity (Unifrac PCoA) and **(B)** alpha diversity (Shannon and Simpson indices). **(C)** Cecum microbiota taxa associated with maternal genotypes (*p* < 0.05, *q* < 0.05) and plotted as relative abundance ratios. **(D)** PICRUSt analysis of the neonatal cecum microbiota plotted as differentially regulated pathways associated with maternal genotype (*p* < 0.05, *q* < 0.05). **(E)** Principal Component Analysis (PCA) plot depicting the clustering of pups based on their cecal SCFA profile. **(F)** Concentration of SCFA present in the cecum of pups normalized by cecal content. Heatmap depicting the relative abundance ratios for the selected taxa in **(G)** cross-fostered pups and **(H)** C-Sec-delivered pups associated with maternal genotypes. Heatmap depicting the selected metabolic pathways associated with maternal genotype in **(I)** cross-fostered pups and **(J)** C-Sec-delivered pups associated with maternal genotype. **(A-D)** Data pooled from two independent litters per maternal genotype; n= 38-26 mice per group. **(E-F)** Data from one litter per maternal genotype; n= 9-10 mice per group. **(G-J)** Data pooled from at least two independent litters per maternal genotype; n= 13-7 mice per group (with the exception of the group of pups born from TCRδ^-/-^ via C-Sec and fostered by a mother of the same genotype, where only one pup survived the procedure). **(C)** Error bars represent Mean ±SD; Statistical analyses for taxonomy and PICRUSt results were performed using the MaAsLin pipeline with Benjamini–Hochberg false-discovery rate (BH-FDR) (q value) *q* < 0.05 and *p* < 0.05 being considered significant (**F)** Box-and-whisker plots display first and third quartiles, and the median; whiskers are from each quartile to the minimum or maximum; Normality of the samples was assessed with D’Agostino Pearson normality test; statistical analysis was then performed using Student’s *t* test or Mann-Whitney test. * *p* < 0.05; *** *p* < 0.001; **** *p* < 0.0001. b., born from.

Taken together, our data led us to hypothesize that the offspring of TCRδ^-/-^ and TCRδ^+/-^ dams are subjected to a distinct neonatal microbial colonization. While no differences were found in bacterial diversity and richness in the lung microbiota from the progeny of TCRδ^-/-^ and TCRδ^+/-^ dams (Figures S8A and S8B), analysis of the microbial composition of the cecal contents evidenced significant, albeit minor, differences in bacterial diversity (Figure 5A) and a decrease in bacterial richness in pups born from TCRδ^-/-^ dams (Figure 5B). No differences in bacterial diversity or richness were found based on the genotype of the progeny (Figure S8C and S8D). Pups born from TCRδ^+/-^ dams displayed significantly increased relative abundances in the genera *Rikenellaceae RC9 gut group, Muribaculaceae* and *Lachnospiraceae UCG-006*. At the family level, the relative abundance of *Rikenellaceae, Muribaculaceae, Oscillospiraceae, Ruminococcaceae*, and *Erysipelatoclostridiaceae* was significantly higher in the offspring of TCRδ^+/-^ dams (Figures 5C and S9C). Pups born from TCRδ^-/-^ dams, on the other hand, displayed an increase in the genus *Lachnoclostridium* (Figures 5C and S8E). Most importantly, to evaluate the relevance of these taxonomy changes, we used the Phylogenetic Investigation of Communities by Reconstruction of Unobserved States (PICRUSt) pipeline to predict the functional metagenome of cecal bacterial communities found in the offspring of TCRδ^-/-^ and TCRδ^+/-^ dams^37^. Several metabolic pathways were predicted to be upregulated in the microbiota of pups born from TCRδ^+/-^ dams. Interestingly, the 20 most significantly different metabolic pathways were enriched for pathways comprising fatty acid biosynthesis and degradation, and fermentation to SCFA (Figure 5D). Corroborating our *in-silico* predictions, analysis of cecum levels of SCFA by gas-chromatography–mass-spectrometry showed a distinct SCFA profile for the progeny of TCRδ^-/-^ and TCRδ^+/-^ dams (Figure 5E), with pups born from TCRδ^+/-^ dams presenting increased levels of pentanoate and hexanoate, and, although not statistically significant, a trend for increased levels of acetate (*p* = 0.0506); no differences were observed in other SCFA measured (Figures 5F and S9F). Altogether, these results indicate that a differential neonatal intestinal bacterial colonization between pups born from TCRδ^-/-^ and TCRδ^+/-^ dams results in changes in the availability of microbial-derived metabolites, ultimately impacting on the first-breath-induced reaction.

Germane to our working hypothesis, given that cross-fostered and C-Sec-delivered pups showed no differences in lung type-2 immunity, we predicted that disruption of neonatal colonization by cross-fostering and C-Sec surgeries also abrogated the differences in bacterial composition and their metabolic potential. Indeed, we found no differences in intestinal bacterial richness in cross-fostered pups (Figure S9A), even though they still presented a significant difference in bacterial diversity (Figure S9B). For C-Sec-delivered pups we observed a more complex picture: the use of the Shannon index revealed no significant differences in bacterial richness between the analyzed C-Sec groups, irrespective of their birth or foster mother (Figure S9C); the Simpson index, however, shows small differences in bacterial evenness depending on the genotype of the foster mother (Figure S9C). Likewise, C-Sec-delivered pups still retained some significant differences in bacterial diversity, again, mostly dependent on the genotype of the foster mother (Figure S9D). As expected, cross-fostering largely homogenized the top 30 most abundant taxa between the offspring of TCRδ^-/-^ and TCRδ^+/-^ dams; C-Sec-delivery, on the other hand, was less consistent but suggested that some of the most represented taxa are acquired vertically after birth and are highly dependent on the genotype of the foster dam (Figure S9E). Taken together, the comparison of the microbiota from cross-fostered and C-Sec-delivered pups indicates that most bacterial communities are transferred postnatally, with some being seeded around birth and persisting even after a perturbation such as cross-fostering. Accordingly, when we focused our analysis specifically on the taxa found to be significantly different between the offspring of TCRδ^-/-^ and TCRδ^+/-^ dams, we observed that cross-fostering completely abrogated those differences (Figure 5G and S10A). Analysis of the microbiota from C-Sec-delivered pups showed that bacteria from the family *Rikenellaceae* and genus *Rikenellaceae RC9 gut group* are transferred during fostering and only by TCRδ^+/-^ dams (Figure 5H and S10B); all the other significantly different bacterial communities, however, were found to be largely similar between groups, with the exception of bacteria from the family and genus *Muribaculaceae*, which were significantly increased in pups born from TCRδ^-/-^ dams via C-Sec and fostered by TCRδ^+/-^ mothers (Figure 5H and S10B). Finally, *in silico* prediction of microbial functionality confirmed that cross-fostering abrogated the differences in the metabolic pathways related to fatty acid biosynthesis and degradation and SCFA production observed in pups born from TCRδ^-/-^ and TCRδ^+/-^ dams (Figure 5I and S10C). Interestingly, C-Sec-delivered pups fostered by TCRδ^+/-^ dams retained the upregulation of pathways controlling fatty acid metabolism, but lost the differences in the pathways related to SCFA production (Figure 5J and S10D). Given that bacteria from the families *Oscillospiraceae, Ruminococcaceae*, and *Erysipelatoclostridiaceae*, and genus *Lachnospiraceae UCG-006* were significantly decreased in the progeny of TCRδ^-/-^ dams but equally distributed among cross-fostered and C-sec-delivered pups, we propose that these taxa are vertically transferred postnatally to the pups from TCRδ^+/-^ dams having a suppressive effect on neonatal lung type-2 immunity. Moreover, our data, together with reports from the literature^38,39^, link these taxa to SCFA production, suggesting that these microbial-derived metabolites may mediate the proposed regulatory gut-lung axis.

### Microbial-derived SCFA prevent exacerbated pulmonary first-breath-induced type-2 inflammation and control post-weaning response to helminth infection

**Figure 6:**
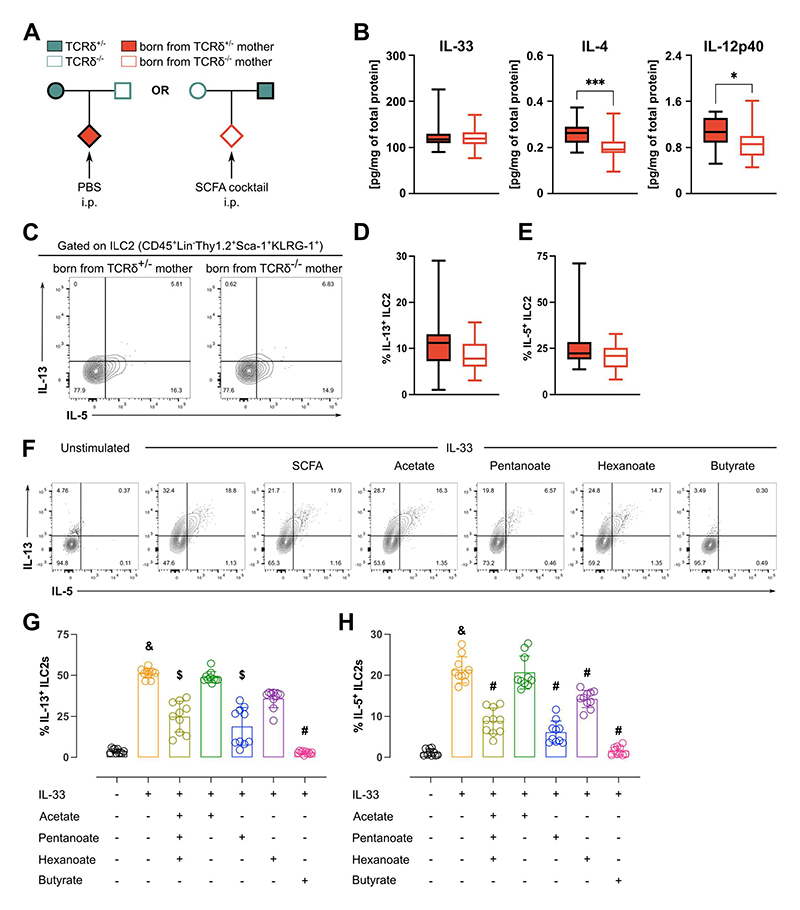
Microbiota-derived SCFA in the offspring suppress exacerbated perinatal type-2 inflammatory responses in the lung. **(A)** Breeding strategy employed to evaluate the role of SCFA in regulating perinatal lung inflammation. **(B)** Concentration of cytokines within the whole lung homogenate normalized by total protein, grouped by maternal genotype. **(C)** Flow cytometry analysis of intracellular IL-5 and IL-13 in ILC2s isolated from the lungs and stimulated *in vitro* for 3h in the presence of PMA, Ionomycin and Brefeldin A. Frequencies of **(D)** IL-13^+^ and **(E)** IL-5^+^ cells within the ILC2 population in the lungs of pups depicted in (A), grouped by maternal genotype. **(F-H)** ILC2s were electronically sorted, plated and res-stimulated for 24h *in vitro* with the conditions depicted. **(F)** Representative FACS plots showing IL-5 and IL-13 production. Quantification of **(G)** IL-13^+^ and **(H)** IL-5^+^ ILC2s. **(B-E)** Data pooled from three independent litters per maternal genotype; n= 17-18 mice per group. **(F-H)** Data pooled from two independent sorting sessions with 5 C57Bl6/J mice pooled per experiment; n= 10 mice per group **(B; D-E)** Box-and-whisker plots display first and third quartiles, and the median; whiskers are from each quartile to the minimum or maximum; Normality of the samples was assessed with D’Agostino Pearson normality test; statistical analysis was then performed using Student’s t test or Mann-Whitney test. * p < 0.05; *** p < 0.001; **** p < 0.0001. **(F-H)** &, *p* < 0.0001 when compared to non-stimulated control; $, *p* < 0.01 when compared to IL-33-stimulated control; #, *p* < 0.0001 when compared to IL-33-stimulated control.

In order to test if exogenous SCFA supplementation was sufficient to suppress the increased type-2 activation observed in the lungs of pups born from γδ T cell-deficient dams, we administered a SCFA cocktail to the offspring of TCRδ^-/-^ dams (Figure 6A). Confirming our hypothesis, administration of the SCFA cocktail to pups born from TCRδ^-/-^ dams abrogated the differences observed in the tissue levels of IL-33, whilst significantly decreasing IL-4 and IL-12p40 concentrations (Figure 6B). Moreover, the treatment also downregulated ILC2 activation and function in the offspring of TCRδ^-/-^ dams, as evidenced by similar frequencies of IL-13^+^ and IL-5^+^ cells (Figures 6C–E). Importantly, administration of the SCFA cocktail via drinking water to TCRδ^-/-^ dams was not sufficient to decrease the frequencies of IL-13- and IL-5-producing ILC2 in their progeny when compared to that of their TCRδ^+/-^ counterparts (Figure S11A–D); lung levels of IL-33 were also still higher in pups born from TCRδ^-/-^ dams (Figure S11E). In order to increase our mechanistic understanding of how SCFAs regulated type-2 immunity, we employed an *in vitro* system to restimulate purified ILC2s with IL-33 in the presence of the SCFA cocktail, its individual components or butyrate as a positive control for ILC2 suppression^40^. Corroborating our *in vivo* data, the addition of the SCFA cocktail to IL-33-stimulated ILC2s decreased the production of IL-13 and IL-5 (Figure 6F–H). Moreover, addition of pentanoate alone was sufficient to recapitulate the effects of the full cocktail (Figure 6F–H); hexanoate, on the other hand, significantly decreases IL-5 production, but not IL-13 (Figure 6F–H). Notably, no differences in cytokine production were observed upon the addition of acetate (Figure 6F–H). Altogether, these findings suggest that bacterial-derived SCFA as inferred from the microbial consortial analysis, in particular pentanoate and hexanoate, regulate the first-breath reaction by directly suppressing ILC2 cytokine production.

**Figure 7:**
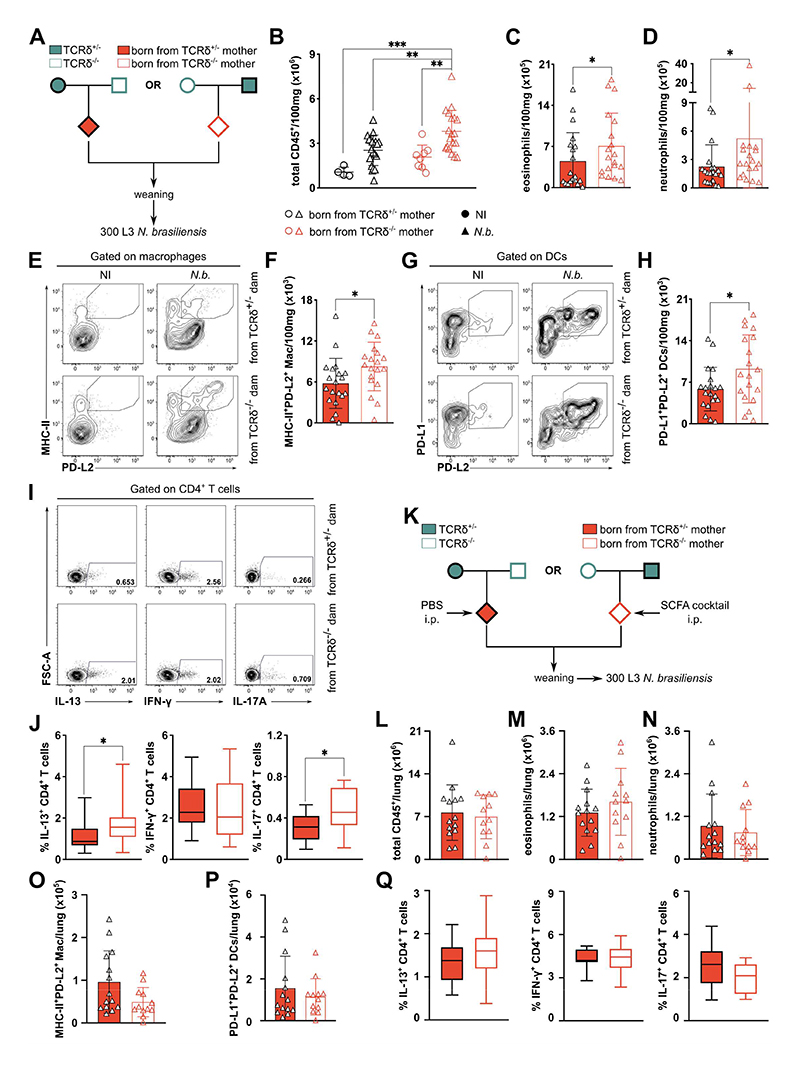
SCFA control exacerbated response to *N. brasiliensis* infection in the offspring of γδ T cell-deficient dams. **(A)** Breeding strategy employed to evaluate the gene-extrinsic and gene-intrinsic roles of γδ T cell depletion on pulmonary immunity. Numbers of **(B)** total leukocytes, **(C)** eosinophils, and **(D)** neutrophils in mice described in (A). **(E)** Flow cytometry analysis of surface MHC-II and PD-L2 expression in lung macrophages (defined as CD45^+^CD64^+^CD24^-^ live cells) **(F)** Numbers of MHC-II^+^PD-L2^+^ macrophages in the lungs. **(G)** Flow cytometry analysis of surface PD-L1 and PD-L2 expression in lung dendritic cells (DCs; defined as CD45^+^CD64^-^CD24^+^CD11c^+^MHC-II^+^ live cells) isolated from the lungs. **(H)** Numbers of PD-L1^+^PD-L2^+^ DCs in the lungs. Flow cytometry analysis of IL-13^+^, IFN-γ^+^ and IL-17A^+^ CD4^+^ **(I)** and their frequencies **(J)** in the lungs. **(K)** Breeding strategy employed to evaluate the role of SCFA on regulation of offspring pulmonary immunity. Numbers of **(L)** total leukocytes, **(M)** eosinophils, **(N)** neutrophils, **(O)** MHC-II^+^PD-L2^+^ macrophages, **(P)** PD-L1^+^PD-L2^+^ DCs in the lungs of mice described in (K). **(Q)** Frequencies of IL-13^+^, IFN-γ^+^ and IL-17A^+^ CD4^+^ T cells in the lungs of mice described in (K). Data pooled from three independent litters per genotype **(B-J)** n= 14-16 mice per group. **(L-P)** n= 12-14 mice per group. **(B-D; F; H; L-P)** Each symbol represents an individual mouse. Error bars represent Mean ±SD. **(J; Q)** Box-and-whisker plots display first and third quartiles, and the median; whiskers are from each quartile to the minimum or maximum. Normality of the samples was assessed with D’Agostino Pearson normality test; statistical analysis was then performed using Student’s *t* test or Mann-Whitney test. * *p* < 0.05; ** *p* < 0.01; *** *p* < 0.001

The increase in first-breath-induced type-2 inflammation in the pups born from γδ T cell-deficient dams, together with our initial results using the *N. brasiliensis* infection model prompted us to further investigate how the offspring of TCRδ^-/-^ dams would respond to a type-2-promoting insult. In order to address this question, mice were infected at the post-weaning period with *N. brasiliensis* (Figure 7A). Although we did not observe differences in lung damage at day 2 or 6 post-infection (p.i.), or in gut worm burden at day 6 p.i. (Figures S12A–C), mice born from TCRδ^-/-^ dams presented an increased inflammatory response to the infection when compared to the offspring of γδ T cell-sufficient dams, characterized by higher numbers of total leukocytes infiltrating the lung (Figure 7B), and enhanced eosinophilia and neutrophilia (Figures 7C and 7D). Surface expression of Programmed Cell Death-Ligand 2 (PD-L2) in macrophages and dendritic cells (DCs) has been reported to occur under strong type-2 conditions^41,42^. Accordingly, we observed increased numbers of PD-L2-expressing macrophages (Figures 7E and 7F) and DCs (Figures 7G and 7H) in mice born from TCRδ^-/-^ dams, when compared to controls. In line with these results, we also found an increase in IL-13^+^ and IL-17A^+^ (but not IFN-γ^+^) CD4^+^ T cells (Figures 7I and 7J) in the progeny of TCRδ^-/-^ dams. When mice were categorized according to their own genotype we observed no differences between TCRδ^-/-^ and TCRδ^+/-^ animals in any of the aforementioned parameters (Figures S12D–I). Taken together, we have found that the offspring of TCRδ^-/-^ dams display stronger type-2 immune responses, irrespective of their own genotype, both in steady-state and upon an infectious challenge.

Next, we sought to evaluate if SCFA supplementation pre-weaning would also be able to suppress the increased inflammatory response during helminth infection observed in the progeny of TCRδ^-/-^ dams (Figures 7A-J). Therefore, we infected with *N. brasiliensis* SCFA-treated mice, born from TCRδ^-/-^ dams, together with vehicle-treated mice, born from TCRδ^+/-^ dams (Figure 7K). Critically, upon SCFA administration the offspring of TCRδ^-/-^ dams showed no differences, when compared to controls, in the numbers of total leukocytes (Figure 7L), neutrophils (Figure 7M), and eosinophils (Figure 7N) infiltrating the lung upon infection. Furthermore, we also found the number of infection-induced PD-L2-expressing macrophages (Figure 7O) and DCs (Figure 7P) to be similar between mice born from TCRδ^-/-^ and TCRδ^+/-^ dams. Finally, after SCFA supplementation, the progeny of TCRδ^-/-^ dams presented similar frequencies of lung IL-13^+^, IL17^+^, and IFN-γ^+^ CD4^+^ T cells in response to *N. brasiliensis* infection, when compared to mice born from TCRδ^+/-^ dams (Figure 7Q). Taken together, our data ascribe the phenotypes observed in the TCRδ^-/-^ progeny to decreased maternal transfer of SCFA-producing bacteria, which beyond increasing first-breath-induced type-2 inflammation, exacerbates inflammatory responses to helminth infection after weaning.

## Discussion

Non-heritable factors, including the microbiome, are perceived as major contributors to systems physiology and immune variation in healthy humans^11,43^. In mice, many changes in cellular and humoral immune responses have been demonstrated to be microbiota-dependent^44,45^. While this makes the design of experiments aimed at evaluating gene-intrinsic effects on a given phenotype more complex, it can be resolved by the use of littermate controls^11^. Despite being considered “gold standard”, this practice it is still not common in biomedical research; in particular, in the field of γδ T cells and infectious diseases, only about 15% of the published studies since the development of TCRδ^-/-^ mice (1993) have used littermate controls over the years (Figure S13A–B), including a notable absence from top-ranked journals (Figure S13C). This may be at the basis of some of the inconsistencies reported over the years regarding the role of γδ T cells in lung immunity.

The fact that mice from the TCRδ^-/-^ colony had a very different fecal microbiota, when compared to their counterparts coming from the TCRδ^+/-^ colony, was striking: first, because the heterozygous breeding was established with founder animals from the TCRδ^-/-^ colony; and second, because both mouse lines were kept under the same selective environmental pressures. Interestingly, Krishnan et al.^25^ reported significant differences in the oral microbiota of TCRδ^-/-^ mice when compared to genetic background-matched C57BL/6J mice. Moreover, using a model for acute depletion of γδ T cells, Wilharm et al.^26^ found that continuous ablation of γδ T cells induces profound changes in the oral microbiome, compared to control (non-depleted) animals. Altogether, these data suggest that γδ T cells – found in abundant numbers at barrier tissues – actively select bacterial commensals and could explain our findings that even after co-housing, TCRδ^-/-^ dams retain differences in cutaneous bacterial communities. This is in line with evidence that co-housing does not equalize niche-specific microbiota within the intestines of genetically identical mice^46^. As we observed persisting differences in cutaneous bacterial communities in TCRδ^-/-^ dams alongside decreased expression of the AMPs *Gpr15l* and *Reg3g*, we propose that γδ T cells may shape skin microbiota through AMP regulation, in a process that is independent of IL-17A and IL-22. Of note, AMPs have already been shown to shape the microbiota^24,47,48^. In our system, this seems to be tissue-specific, as we did not observe differences in the microbiota or AMP expression in the female reproductive tract of TCRδ^-/-^ mice. Importantly, GPR15L has been shown to be critical for the maintenance of dendritic epidermal T cells (DETCs; Lahl et al., 2014), a major γδ T cell population in the skin, and disruption of this axis in GPR15^-/-^ mice leads to skin dysbiosis^49^.

Previous analysis of human mother-infant pairs corroborates our results, showing that maternal skin and vaginal strains transferred perinatally start the earlier colonization, followed by gut-derived strains^50^. the results we obtained from our cross-fostering and C-sec experiments indicate that most bacterial communities found to be increased in the offspring of TCRδ^+/-^ dams were transferred post-birth. Furthermore, although some metabolic pathways remained different between the pups delivered by C-Sec from TCRδ^-/-^ or TCRδ^+/-^ dams, we observed an equalization of the pathways associated with the production of SCFA. Thus, our results seem to indicate that the period comprising delivery and the few following days are crucial not only to determine the microbiota composition but also its functional capacity. While our results do not directly show which maternal compartment provides the pioneering bacterial communities, our data suggest that the skin of TCRδ^-/-^ and TCRδ^+/-^ dams display differences that may influence neonatal gut colonization and ultimately regulate the first-breath-induced pulmonary reaction.

Even though rodent pulmonary immune system development starts during fetal life^51^, the main changes occur during the first two weeks of life. Changing from a fluid-filled to an air-filled environment entails sustained basal type-2 immune responses – a critical feature to keep organ function during this heavy remodeling phase^9,10^. Our results indicate that early-life gut-colonizing microbes and their metabolites are able to fine-tune this lung type-2 activation, indicating that the process of IL-33 release by airway epithelial cells involves more than physical stress^10^. To this date little was known about the effects of pentanoate and hexanoate on type-2 immune responses. Reports in the literature indicate that pentanoate can present both suppressive and enhancing effects on lymphocytes; either by reducing IL-17 production in CD4^+^ T cells^52^ or inducing IFN-γ production in CD8^+^ T cells^53^. Our *in vivo* and *in vitro* approach demonstrated that pentanoate (and to a lesser extent hexanoate) is also able to directly suppress ILC2 activation and cytokine production. In addition, it is important to note that for the intestinal immune system the action of SCFA seem to occur at the time of weaning^3^. In our study, however, we show that lung immunity is affected by microbiota-derived SCFA before weaning, and *in utero* transfer of SCFA was not responsible for the regulation of lung type-2 immunity in the offspring of TCRδ^-/-^ dams. These findings underscore the discrete maturation time windows for the immune cells residing within different tissues and the context-specific effects of SCFA.

While nutrition, especially during pregnancy and breast feeding, has a major impact on early microbiota composition and offspring physiology^7,12,54^, our study highlights the importance of microbiota transmission from mother-to-infant at the perinatal stage^50^ to the establishment of gut microbiota functionality, which conditions the physiology of a distant organ, the lung, undergoing major immune remodeling during early life.

### Limitations of the study

Our data suggest that maternal γδ T cells regulate postnatal microbial colonization and microbial-derived metabolite availability in the offspring, ultimately impacting the pulmonary immune system development. Even though we observed the presence of different bacterial taxa in the skin of TCRδ^-/-^ and TCRδ^+/-^ dams even after co-housing, together with tissue-specific differences in the expression of AMP genes, we could not pinpoint the mechanism by which γδ T cells might change microbiota composition. Moreover, our data does not allow us to fully determine from which maternal compartment the pioneering bacterial communities are coming from. As γδ T cells are found in relatively high numbers in the female reproductive tract, it was somewhat surprising to observe no differences in either the microbiota or the expression of AMPs in vaginal tissue from TCRδ^-/-^ and TCRδ^+/-^ dams. However, a caveat is that the vaginal tissue used for both 16S sequencing or gene expression analysis was not obtained from pregnant females just before birth. Maternal vaginal microbiota changes significantly during gestation, with a particularly dramatic change happening during the late phases of pregnancy and at birth^55^. Thus, raising the possibility that the absence of γδ T cells might differentially impact the pre-partum changes in the microbiota. Finally, future studies should address the temporal scale that γδ T cells (and other immune cells) require to stably modify the microbiota.

## Star Methods

### Resource Availability

### Lead contact

Further information and requests for resources and reagents should be directed to and will be fulfilled by the lead contact, Bruno Silva-Santos (bssantos@medicina.ulisboa.pt).

### Materials availability

This study did not generate new unique reagents.

### Data and code availability

16S rRNA amplicon sequencing dataset with processed files and the entire details of used samples can be downloaded using the following link: https://figshare.com/s/77865dbbf0daab588b8d (DOI: 10.6084/m9.figshare.15121245). Any additional information required to reanalyze the data reported in this paper is available from the co-author, Bahtiyar Yilmaz (bahtiyar.yilmaz@unibe.ch), upon request. This paper does not report original code.

## Experimental Model And Subject Details

### Mice and breeding setups

C57BL/6J (B6) mice were purchased from Charles River Laboratories, C57BL/6J.TCRδ^-/-^ (referred to as TCRδ^-/-^, hereon in) were purchased from The Jackson Laboratory. C57BL/6J.JHT (referred as JHT) mice were obtained from Instituto Gulbenkian de Ciência (Oeiras, Portugal). All the mouse lines were bred and maintained in the specific pathogen-free animal facilities of Instituto de Medicina Molecular João Lobo Antunes (Lisbon, Portugal), with the exception of IL-22^-/-^, which were generated and acquired from Jean-Cristophe Renauld, and bred in-house at The University of Manchester. After reaching sexual maturity (at around 8 weeks) TCRδ^-/-^ females were crossed with TCRδ^+/-^ males (obtained from the cross of TCRδ heterozygous animals derived, in turn, from a TCRδ^-/-^ x B6 breeding), and vice-versa; the pups obtained from the aforementioned crossings were kept with the parents until weaning, to be later divided by sex. All the pups were used at PN16±2, unless stated otherwise.

### Study approval

All experiments performed in Lisbon involving animals were done in compliance with the relevant laws and institutional guidelines and were approved by local and European ethic committees. All animal experiments were performed in Manchester are in accordance with the UK Animals (Scientific Procedures) Act of 1986 under a Project License (70/8548) granted by the UK Home Office and approved by the University of Manchester Animal Welfare and Ethical Review Body. Euthanasia was performed by asphyxiation in a rising concentration of carbon dioxide.

## Method Details

### Cross-fostering and C-Sec surgeries

For cross-fostering experiments, mice were time-mated. Briefly, female mice were put in a cage containing the bedding of a cage containing male animals for a period of 48h. After this period of time, 1 or 2 females were introduced to a single-housed male. The following day, the presence of vaginal plugs (considered as E0.5) was recorded and males were transferred into a new cage. In some cases, the animals were left together for 2 days. Subsequently, pups born from a TCRδ^-/-^ dam were swapped with those from a TCRδ^+/-^ dam at day 1 post-birt (and vice-versa), respecting litter sizes (differences of 3 pups maximum).

For C-Sec experiments TCRδ^+/-^ and TCRδ^-/-^ females were bred with males from the opposing genotype in two groups, with an interval of 1 day between them. The group of females giving birth first was then defined as the surrogate mothers. Between days 18.5 and 19.5 the females from the second group underwent C-Sec surgery. Briefly, females are euthanized; the uterus is removed and submerged in iodine. Pups are taken out from the uterus on top of a sterile heated plate, cleaned and reanimated. When presenting regular respiratory movements, pups are put together with surrogate dams (from the same genotype of their own dam) and their progeny.

### Cell preparation, flow cytometry, and analysis

For cell surface staining, single-cell suspensions were incubated in presence of anti-CD16/CD32 (eBioscience) with saturating concentrations of combinations of the mAbs listed above. Lungs were dissected and tissue was cut into pieces, then digested with collagenase D (0.66 mg/ml; Roche) and DNase I (0.10 mg/ml) (Sigma-Aldrich) in RPMI 1640 containing 5% Fetal Bovine Serum (FBS) at 37°C for 30 min. For lamina propria cell preparation, small intestines were dissected, washed in ice cold PBS and Peyer’s patches were excised. The organ was then cut into pieces and incubated with EDTA 0.05M at 37°C for 20 min; cells were then washed and passed through a 100-m cell strainer and then digested as the lungs. Single cells were isolated by passing the tissue through a 40-μm cell strainer, followed by a 70% / 20% Percoll (Sigma-Aldrich) gradient and 30-min centrifugation at 2400 rpm. Leukocytes were recovered from the interface, resuspended, and used for further analyses.

For intracellular cytokine staining, cells were stimulated with PMA (phorbol 12-myristate 13-acetate) (50ng/mL) and ionomycin (1μg/mL), in the presence of Brefeldin A (10μg/mL) (all from Sigma) for 3h to 4h at 37°C. Cells were stained for the identified above cell surface markers, fixed 30min at 4°C and permeabilized with the Foxp3/Transcription Factor Staining Buffer set (eBioscience) in the presence of anti-CD16/CD32 (eBioscience) for 10min at 4°C, and finally incubated for 1h at room temperature with identified above cytokine-specific Abs in permeabilization buffer. Cells were analyzed using FACSFortessa (BD Biosciences) and FlowJo software (Tree Star).

For cell sorting of lung interstitial macrophages (IM), lung cell suspensions were prepared and immuno-stained as described above. IM were defined as CD45^+^CD64^+^CD24^-^CD11b^+^CD11c^-^ live cells, and then electronically sorted on a FACSAria (BD Biosciences) into sterile RNase-free eppendorfs containing 200μL of lysis buffer (Qiagen), for posterior mRNA isolation. For cell sorting of ILC2s, lung cell suspensions were prepared and immuno-stained as described above. ILC2s were defined as CD45^+^Lin^-^Thy1.2^+^CD127^+^ST2^+^ live cells.

### In-vitro ILC2 stimulation

To maximise cell yield for sort-purification, mice were injected intraperitoneally with 1μg of recombinant IL-33 (BioTechne) on day 0, 2, 4 and 6. Mice were culled on day 7, lungs were then processed and stained as described before for cell sorting.

Sorted ILC2s were plated at a density of 70000 cells/well (round bottom plate) in 200uL of GlutaMAX media (Gibco™) (supplemented with 10% FBS, 1% MEM Non-Essential Amino Acids Solution (100X) (Gibco™), 1% Penicillin-Streptomycin (Sigma), 1% Sodium pyruvate solution 100mM (Sigma), and β-mercaptoethanol (Sigma)) with IL-7 (25ng/mL, Peprotech). Cells were stimulated with IL-33 (20ng/mL, BioTechne) plus the individual SCFA acetate (0.25mM), pentanoate (0.25mM), hexanoate (0.25mM) or Butyrate (0.25mM) or a cocktail containing the first three SCFA (acetate, 0.25mM; pentanoate, 0.125mM; hexanoate, 0.125mM). Cells were incubated for 24h at 37°C in the presence of CO2, stained intracellularly for IL-13 and IL-5 and analyzed using FACSFortessa (BD Biosciences).

### Immuno-detection of cytokines in lung tissue samples

Lung tissue was homogenized in 2 mL sterile tubes containing PBS plus a cocktail of protease inhibitors (cOmplete ULTRA tablets, Mini; Roche) and 1mm of diameter zirconia/silica beads (BioSpec Products), with the help of a tissue homogenizer. The homogenate was centrifuged in order to remove cellular debris and beads, and stored at -80ºC until usage. Cytokine concentrations were assessed using LEGENDplex™ Mouse Th1/Th2 and Mouse Cytokine Panel 2 kits – both bead-based assays that use the principles of sandwich ELISA to quantify soluble analytes using a flow cytometer (Biolegend) – according to manufacturer’s instruction. Cytokine concentrations were normalized using total protein concentration in tissue homogenate, determined in a NanoDrop spectrophotometer (ThermoFisher).

### Immuno-detection of antibodies in serum samples

Blood was collected from the cheek pouch in adults, and from decapitation in pups. Serum samples were then stored at -20ºC until usage. Determination of immunoglobulins isotypes and serum concentrations were assessed using LEGENDplex™ Mouse Immunoglobulin Isotyping Panel (Biolegend) according to manufacturer’s instruction.

### RNA isolation, cDNA preparation and real-time PCR

mRNA was prepared from tissue homogenates using RNeasy^®^ Mini Kit (Qiagen). Reverse transcription was performed with random oligonucleotides (Invitrogen) using Moloney murine leukemia virus reverse transcriptase (Promega) for 1 hour at 42 °C. Relative quantification of specific cDNA species to endogenous reference *Hprt, Actb* or *β2microglobulin* was carried out using SYBR on ABI ViiA7 cycler (Applied Biosystems). The C_T_ for the target gene was subtracted from the average C_T_ for endogenous references, and the relative amount was calculated as 2^−ΔCT^.

### N. brasiliensis *infection*

*N. brasiliensis* was maintained by serial passage through Sprague-Dawley rats, as previously described^57^. Third-stage larvae (L3) were washed ten times with sterile PBS prior to subcutaneous infection of 300 L3s per mouse. On day 6 post-infection (p.i.) lungs were excised and processed accordingly to the analysis to be conducted.

### Histology and histopathological analysis

Lung samples were fixed in 10% neutral buffered formalin and processed for paraffin embedding, sectioned and stained with Hematoxylin-Eosin. Each slide was digitalized using a NanoZoomer-SQ Digital slide scanner. To quantify emphysema like damage, we used the Linear Mean Intercepts (LMI) method. After digitalization of each lung slide, between 10 and 20 non-overlapping pictures, of each lung, were taken. The pictures were analyzed using ImageJ software. For each picture, 6 horizontal lines were drawn. For the LMI calculation, the length of the line was multiplied by the number of pictures taken and the number of lines in one picture, and then divided by the total number of times a line intercepted an alveolus.

### Antibiotic treatment and SCFA supplementation

TCRδ^-/-^ and TCRδ^+/-^ female and male adult mice were maintained with a mixture of antibiotics (5mg/ml of streptomycin, 1mg/ml of ampicilin, 1mg/ml of collistin, and 0.5mg/ml of vancomycin; Sigma Aldrich) in the drinking water for at least three weeks prior the start of the breedings, and then crossed as already described. Antibiotic treatment was kept for the whole duration of pregnancy and fostering.

For direct supplementation of SCFA, 50μL of a cocktail containing acetate (2mg/mL), pentanoate (0.4mg/mL) and hexanoate (0.35mg/mL) was administered i.p. 3 times/week, starting at PN7 and finishing at PN13 for experiments analyzing the steady-state lung; and starting at PN14 and finishing at PN20 for experiments analyzing the response to *N. brasiliensis* infection. In both cases controls were injected with PBS. When given to dams, a SCFA cocktail containing acetate (50mM), pentanoate (37.5mM) and hexanoate (12.5mM) was added to the drinking water at day 2±1 after the litter was born and kept during the fostering period.

### Microbiota profiling via 16S rRNA gene sequencing

Samples were collected in sterilized surfaces using autoclaved materials. Fecal microbiota was assessed from two to three fecal pellets per animal. Skin microbiota was assessed upon removal of one ear per animal. Vaginal microbiota was assessed from the vaginal wash with 30μL of sterile PBS. Lung and cecum mucosa associated microbiota were assessed from the whole excised organs. Immediately after collection, samples were snap-frozen in liquid nitrogen and stored at −80°C until microbial DNA extraction. Before extraction, samples were homogenized in 2mL sterile tubes containing 1mm of diameter autoclaved zirconia/silica beads (BioSpec Products), with the help of tissue homogenizer. Microbial genomic DNA was extracted using the QIAamp® Fast DNA Stool Mini kit (Qiagen). The 16S rRNA V4 amplicons and ITS1-spanning amplicons were both generated using the following Earth Microbiome Project benchmarked protocols. Amplicons were then sequenced using on a 2x250bp PE mode Illumina MiSeq platform at Instituto Gulbenkian de Ciências (IGC) Genomics Unit (Oeiras, Portugal).

Raw sequences obtained from 2 independent Illumina MiSeq runs were initially pooled and analyzed in the QIIME pipeline version 2 using custom analysis scripts for analysis on the UBELIX Linux cluster of the University of Bern (High-Performance Computing, University of Bern, Bern, Switzerland). Amplicon sequencing variants were assigned using Greengenes (13_8) databases with the *feature-classifier classify-sklearn* function for 97% sequence identity threshold^58^. The taxonomy, rep-seqs, and rooted-tree files generated in QIIME2 were called out in the phyloseq pipeline in R. Calculation and plotting of the alpha diversity (Simpson and Shannon index), beta diversity (Bray-Curtis dissimilarities on NMDS plot), and statistical analysis using Adonis test were performed using phyloseq pipeline^59–62^. The multivariate analysis by linear models (MaAsLin2) was used to find associations between metadata and microbial community abundance (https://huttenhower.sph.harvard.edu/maaslin/)^63^. Taxa that were present in ≥30% of the samples and had >0.001% of total abundance were set as the cut-off values for further analysis. After Benjamini-Hochberg’s false discovery rate correction, the adjusted p-value (<0.05 or <0.2) was considered significant.

The prediction of metagenome functional content was performed using 16S sequencing data in the Phylogenetic Investigation of Communities by Reconstruction of Unobserved States (PICRUSt2) pipeline^64^. PICRUSt2 predictions were categorized as levels 1–3 and further into Kyoto Encyclopedia of Genes and Genomes pathways.

### SCFA measurement

Sample analysis was carried out by MS-Omics (www.msomics.com) as follows. Samples extracted by adding ultrapure water to the cecum (100μL to samples with less than 30mg and 150μL to samples with more). The filtered cecum water was further extracted by addition of deuterium labelled internal standards and mixing with MTBE directly in the injection vial. All samples were analyzed in a randomized order. Analysis was performed using a high polarity column (ZebronTM ZB-FFAP, GC Cap. Column 30m x 0.25mm x 0.25μm) installed in a GC (7890B, Agilent) coupled with a quadropole detector (5977B, Agilent). The system was controlled by ChemStation (Agilent). Raw data was converted to netCDF format using Chemstation (Agilent), before the data was imported and processed. The raw GC-MS data was processed by software developed by MS-Omics and collaborators. The software uses the powerful PARAFAC2 model and can extract more compounds and cleaner MS spectra than most other GC-MS software.

### Literature review

A search was performed in the PubMed data base in May/2019, using the keywords “gamma delta T cells AND infection” and “gamma-delta T cells AND infection”. Studies employing TCRδ^-/-^ mice were then divided regarding the use of littermates or genetic background-matched mice as controls. Journals were divided by their impact factor at the moment of the literature search.

## Quantification And Statistical Analysis

### Statistical Analysis

In most experiments 3 independent litters were pooled together, unless stated otherwise. D’Agostino-Pearson normality test was applied to assess the normality of the populations analyzed; statistical significance of differences between populations was, then, assessed with the Student’s t-test or by using a two-tailed nonparametric Mann-Whitney U test, when applicable. For grouped statistical analysis, Two-Way ANOVA was employed. The p values <0.05 were considered significant and are indicated on the figures.

## Supplementary Material

Supplementary Figures
