# Supplementary figures and images for: Maternal γδ T Cells Shape Offspring Pulmonary Type-2 Immunity In A Microbiota-Dependent Manner
Source: Cell Rep. Author manuscript; Available in PMC 2024 Feb 14. (PMC7615642; doi:10.1016/j.celrep.2023.112074)

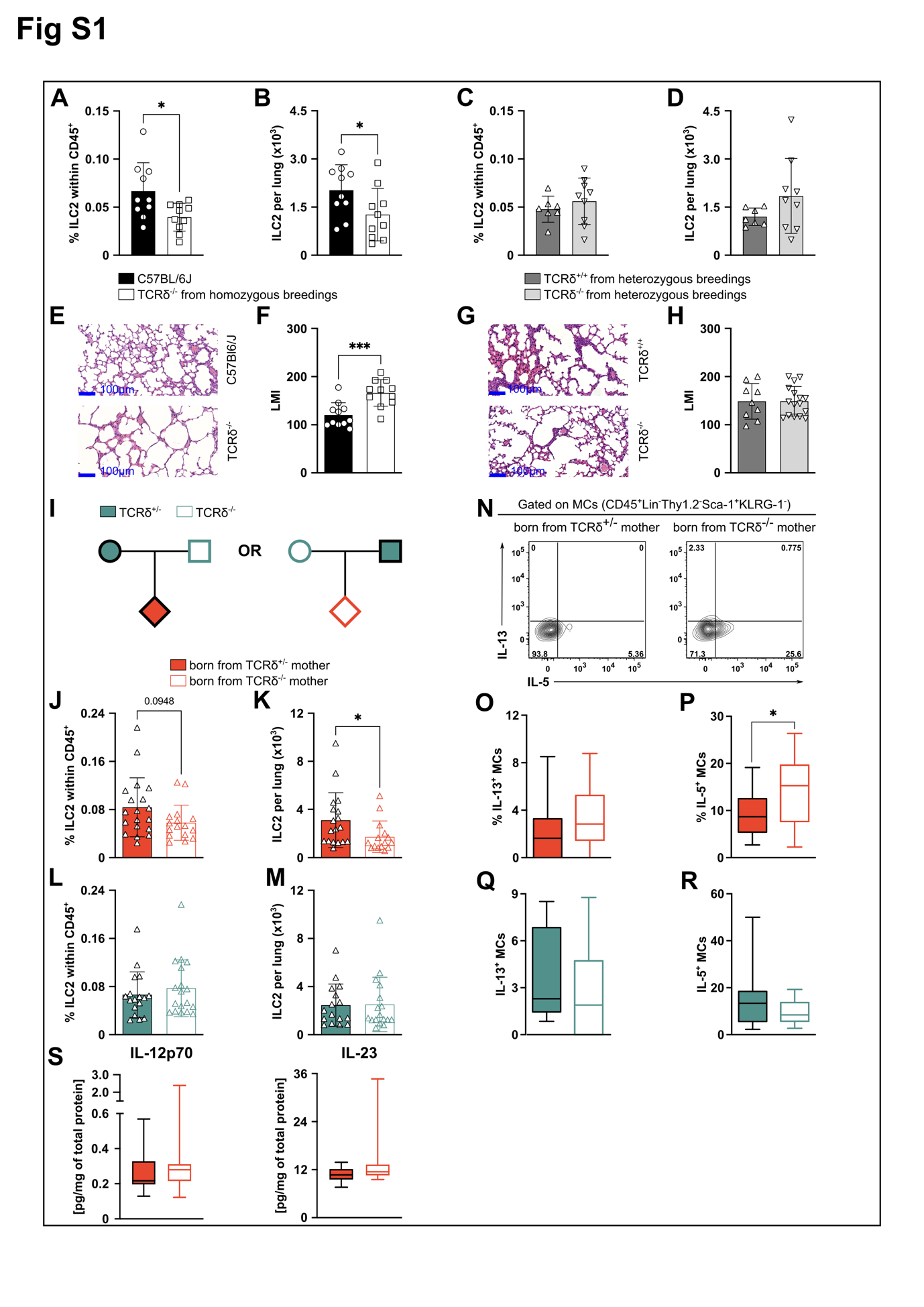


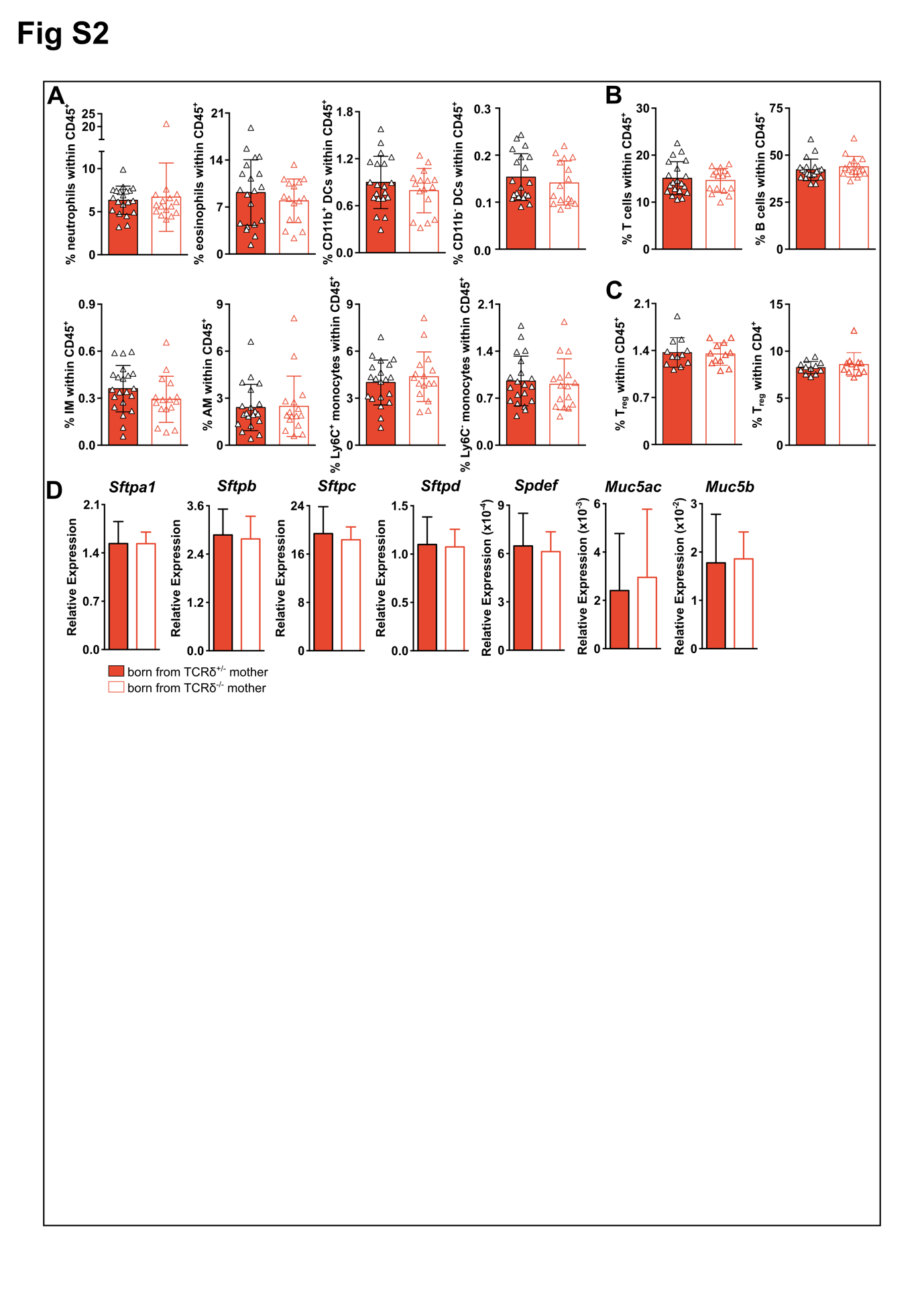


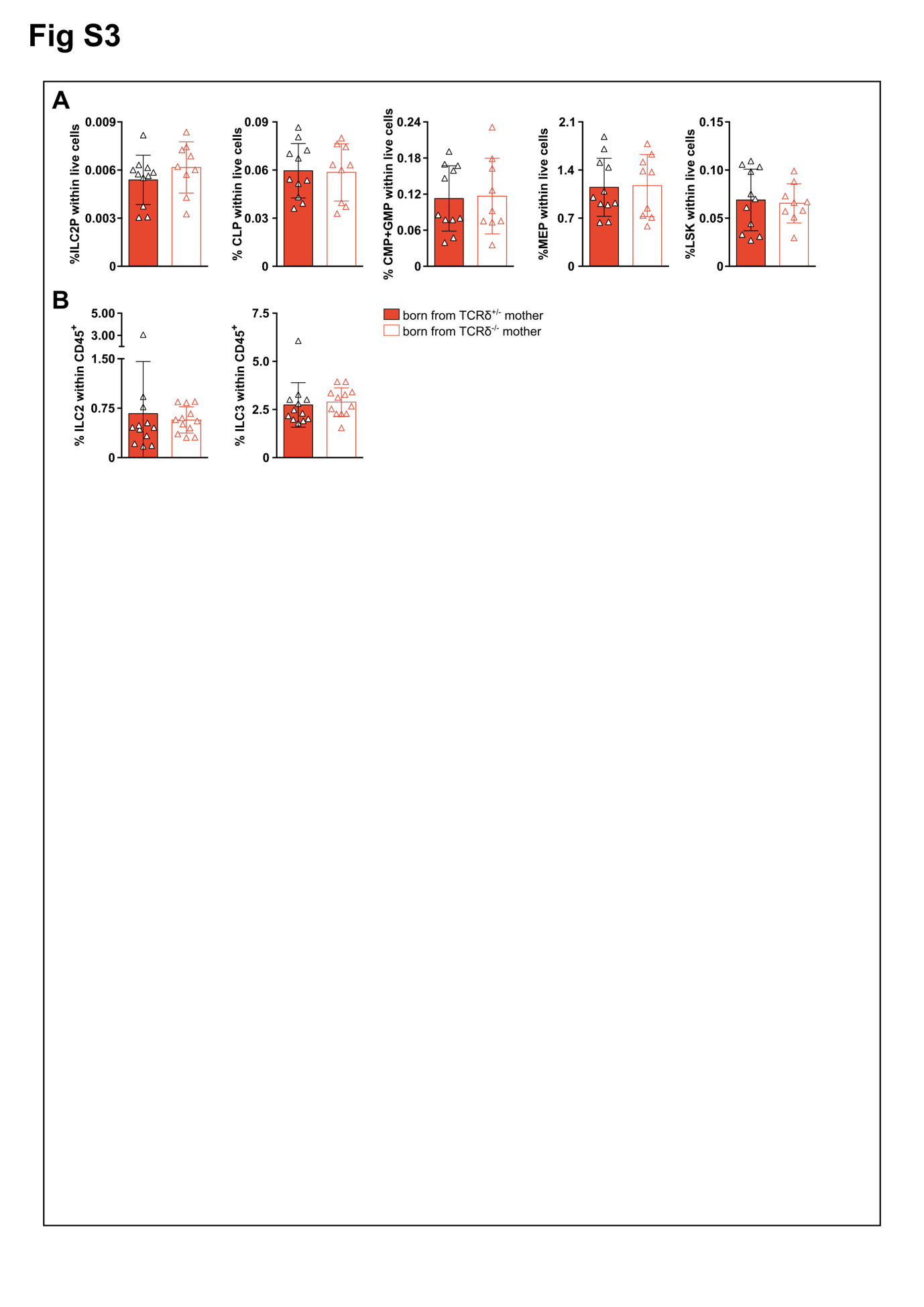


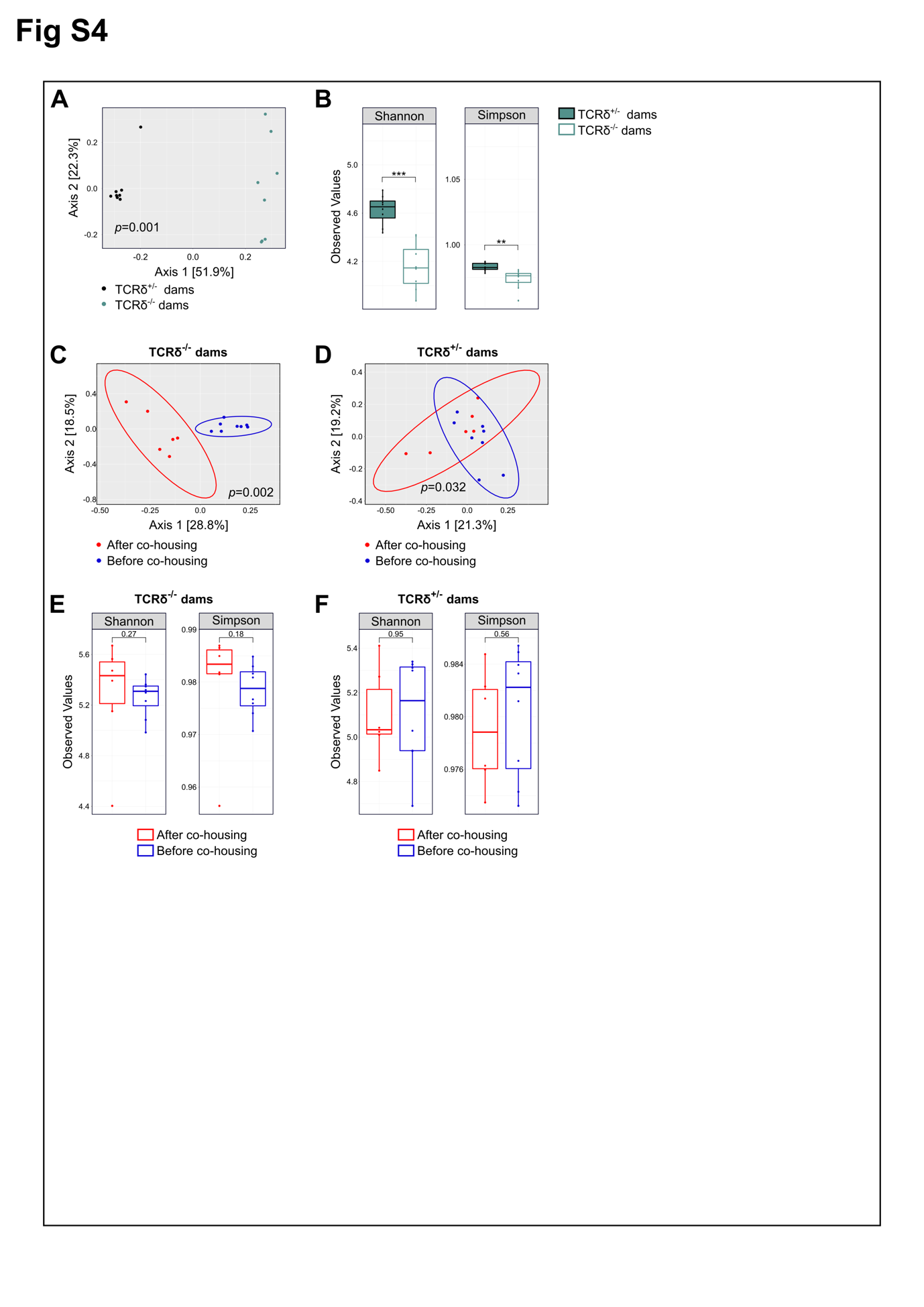


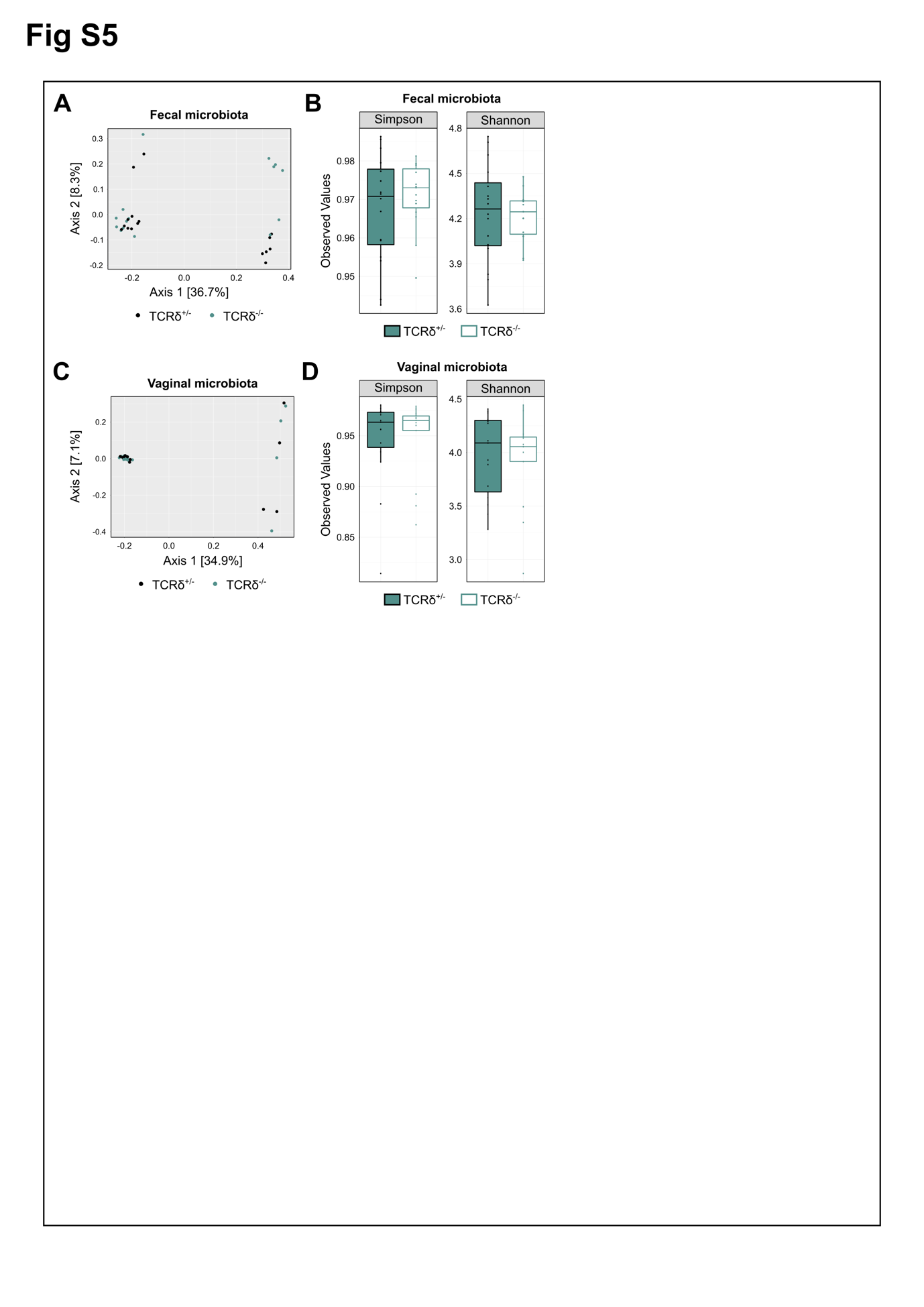


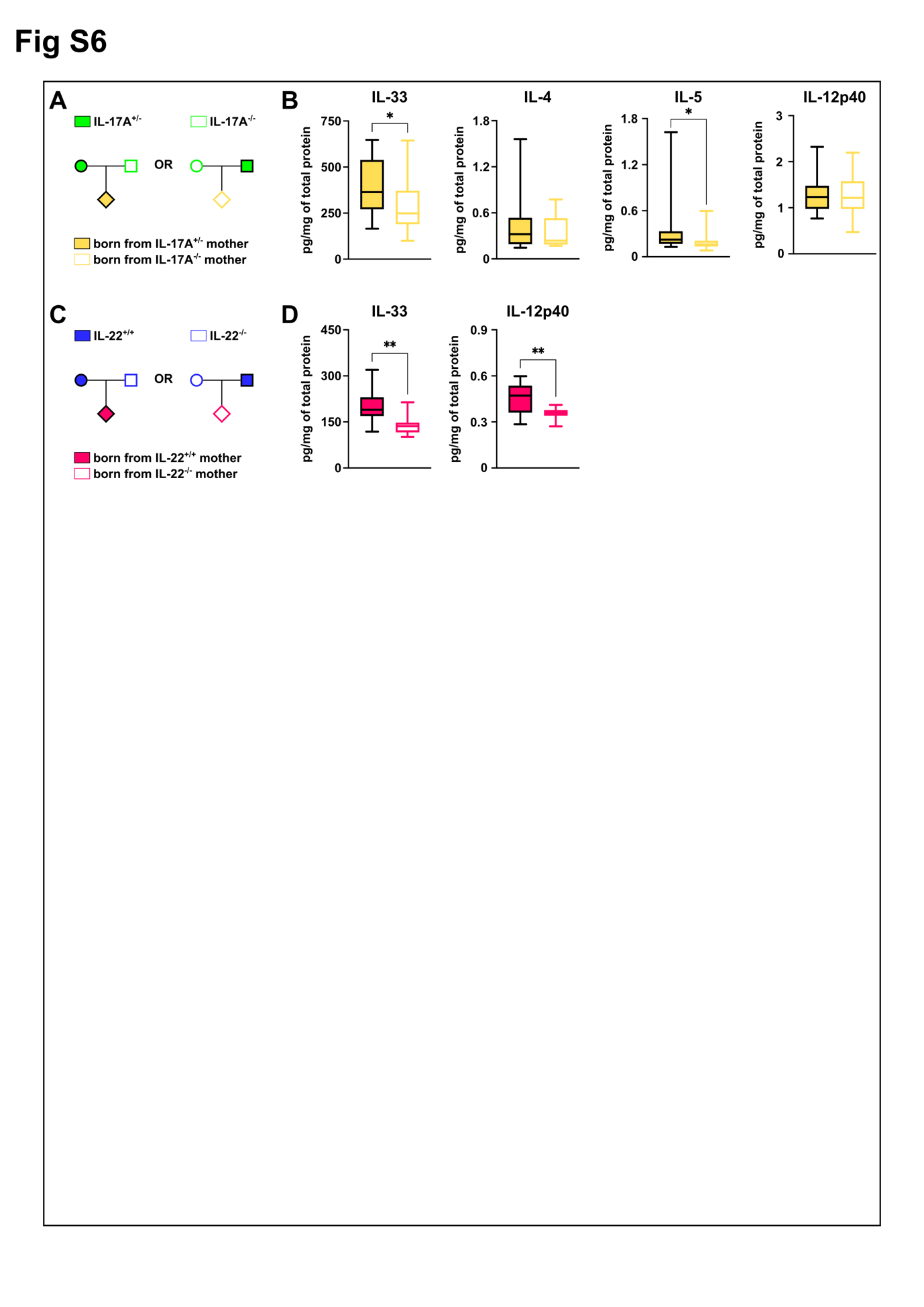


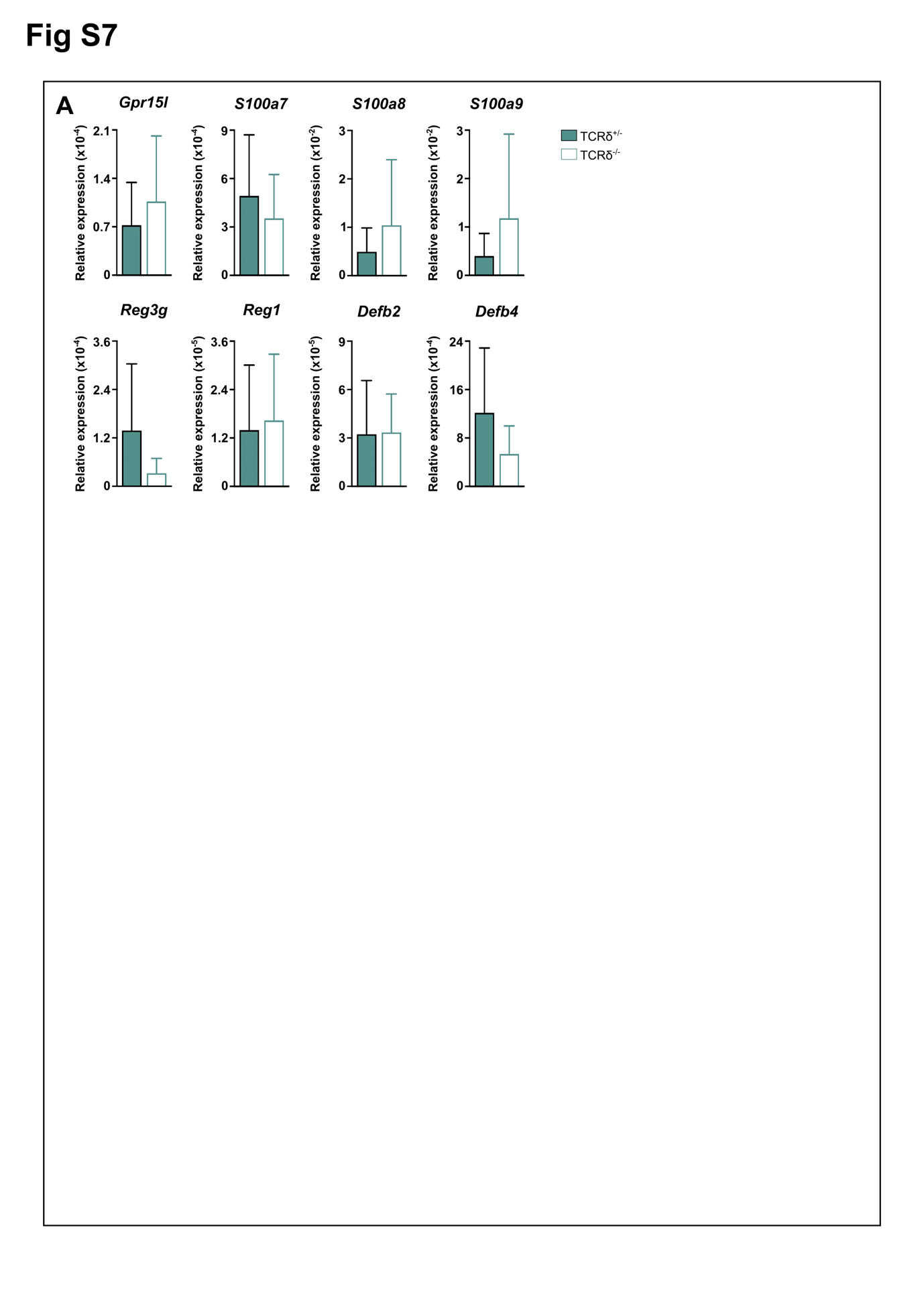


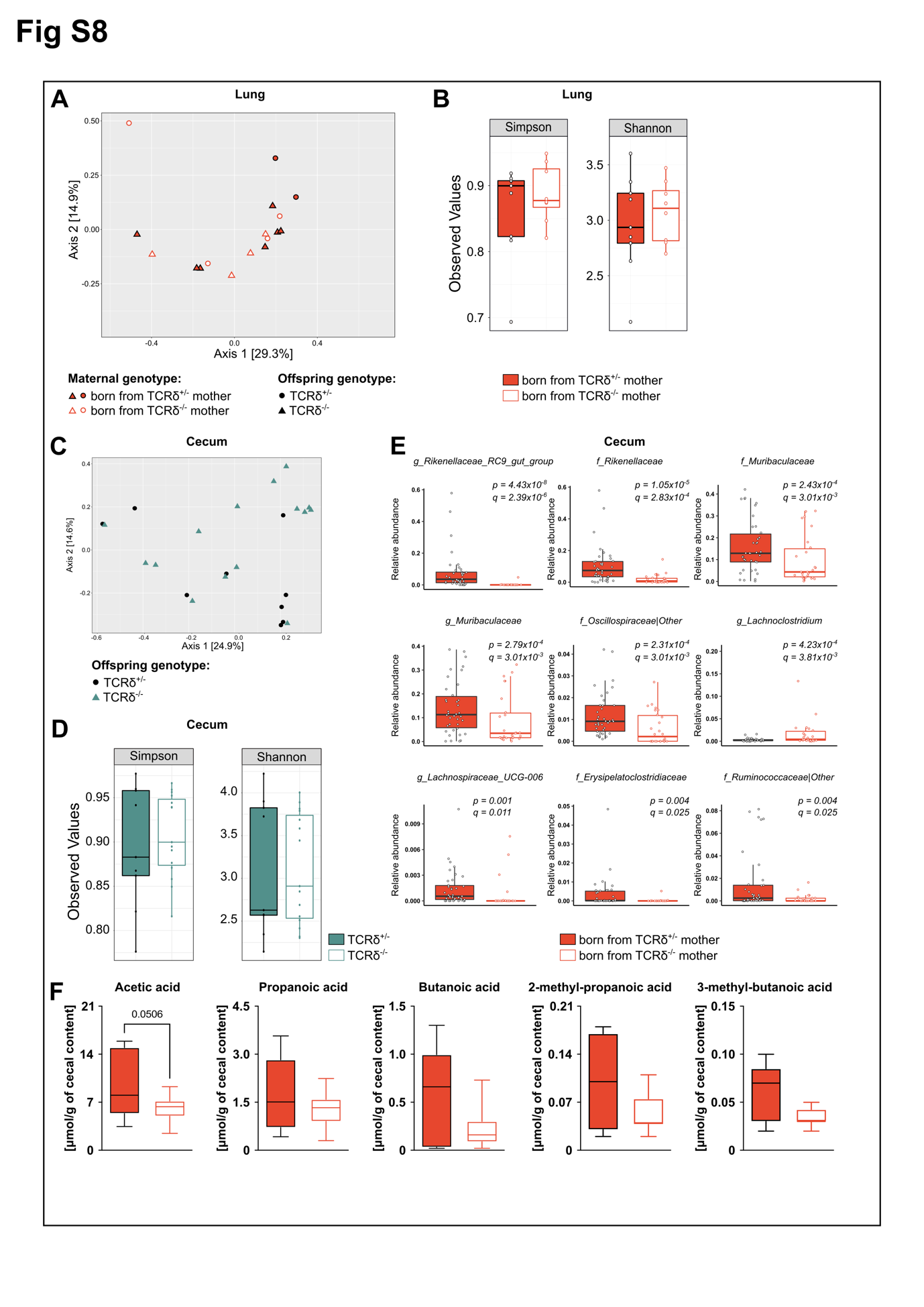


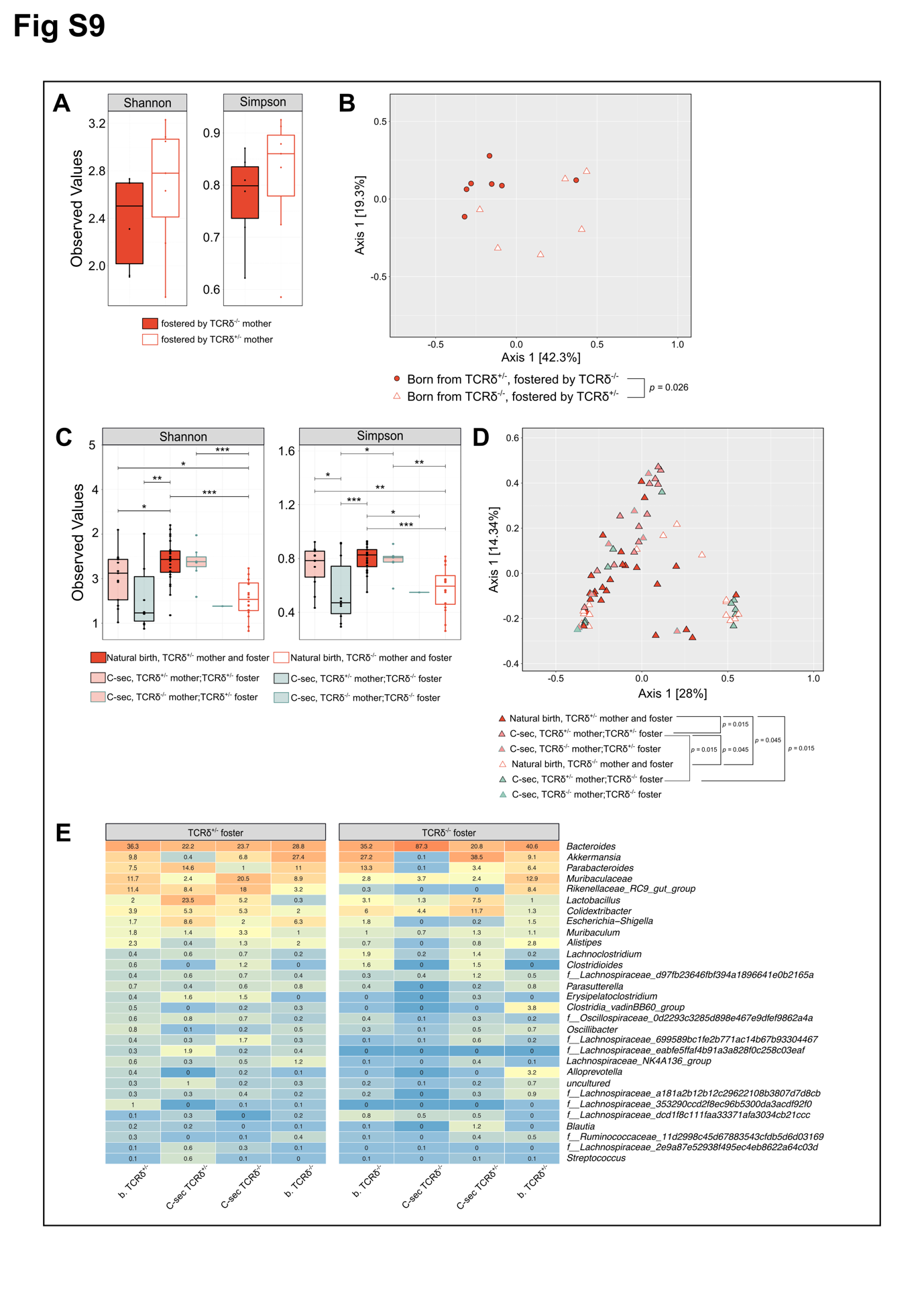


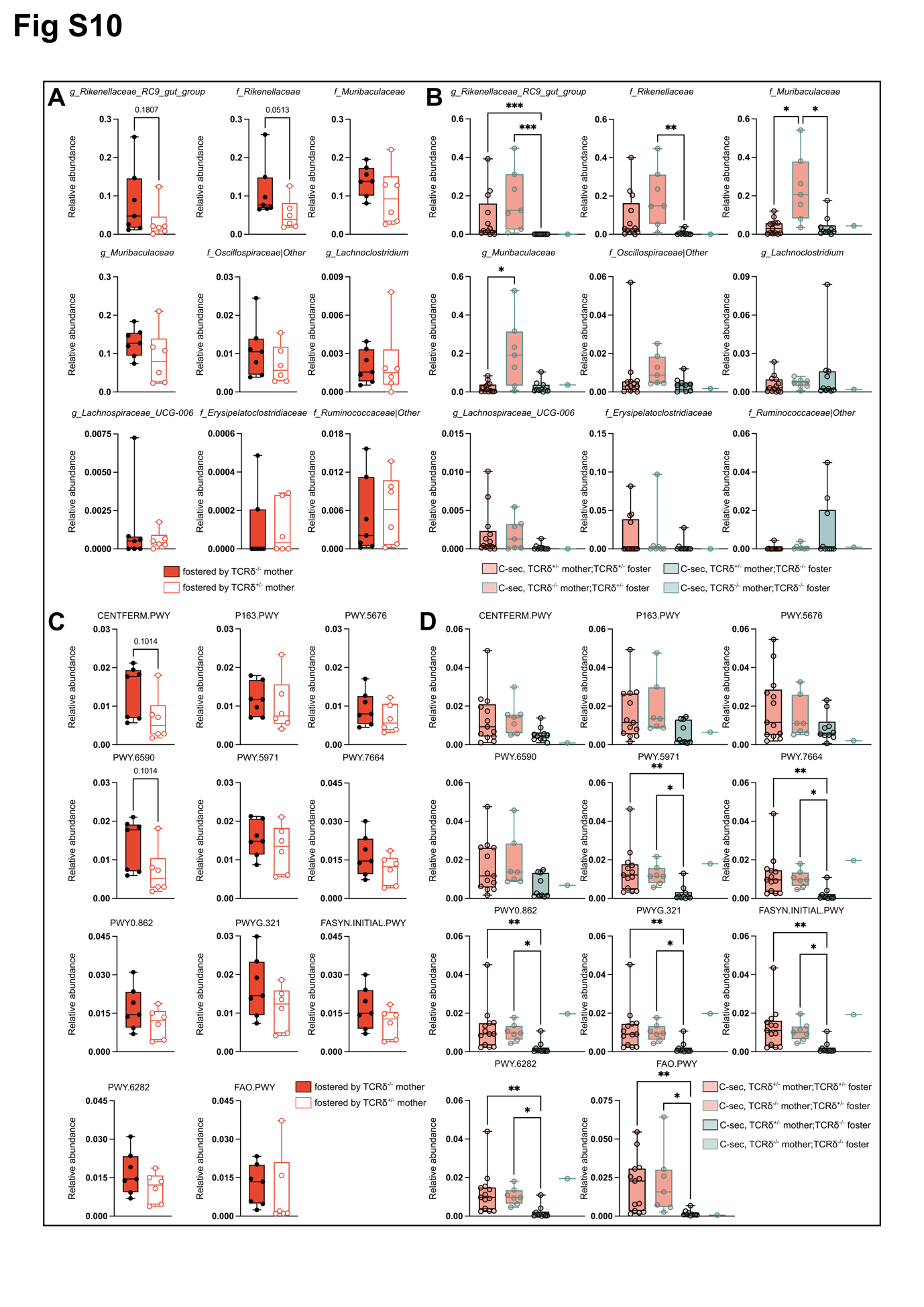


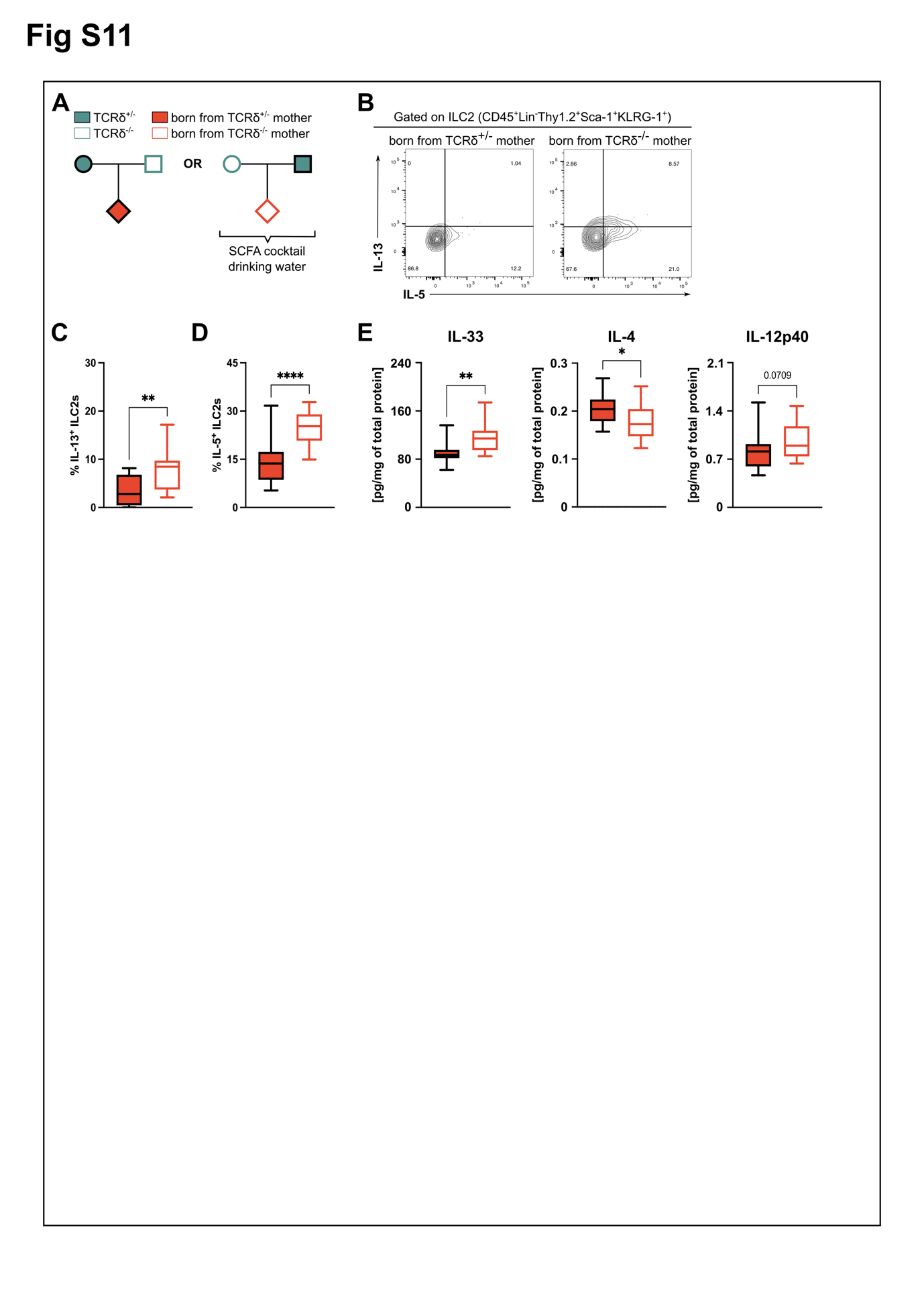


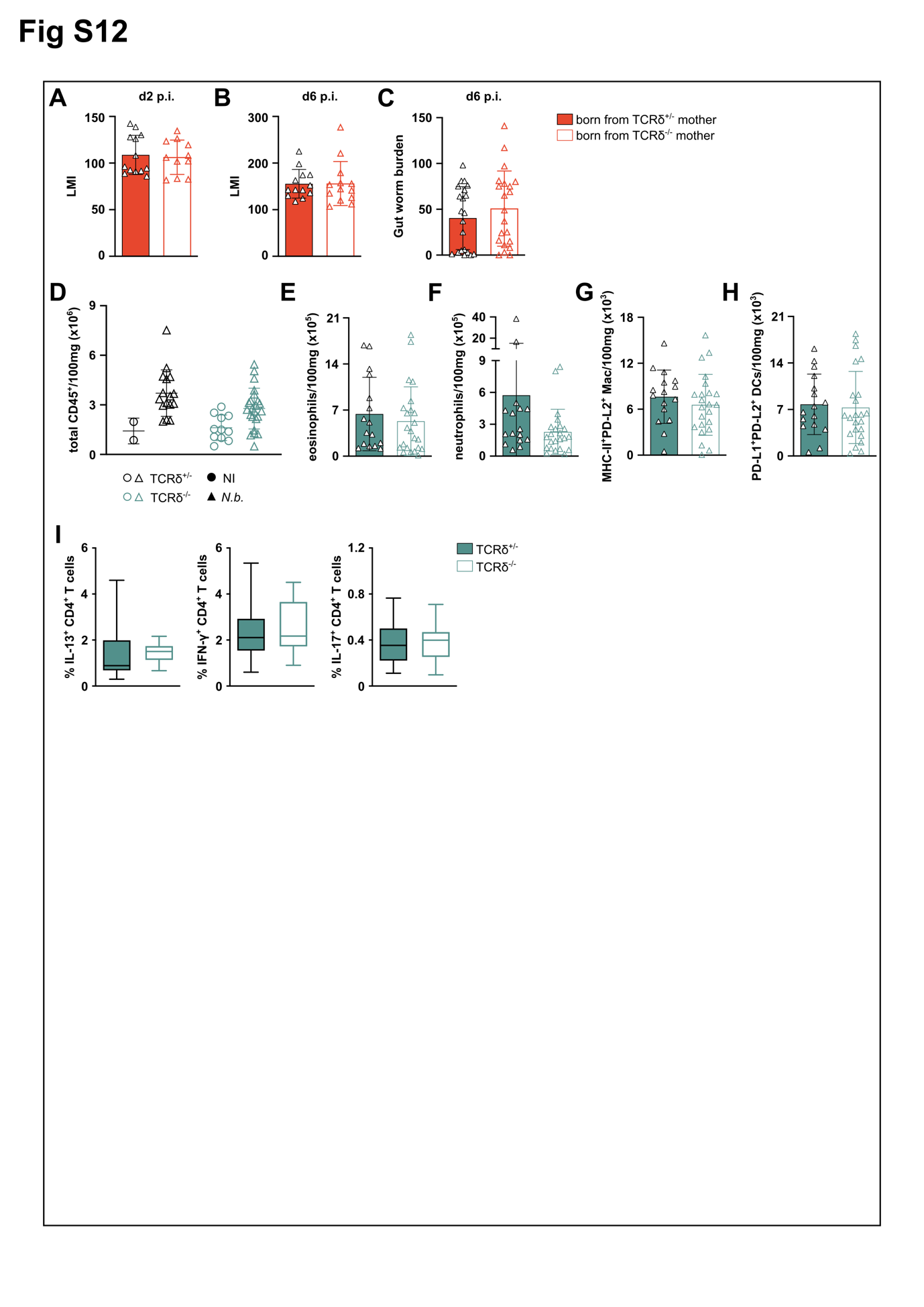


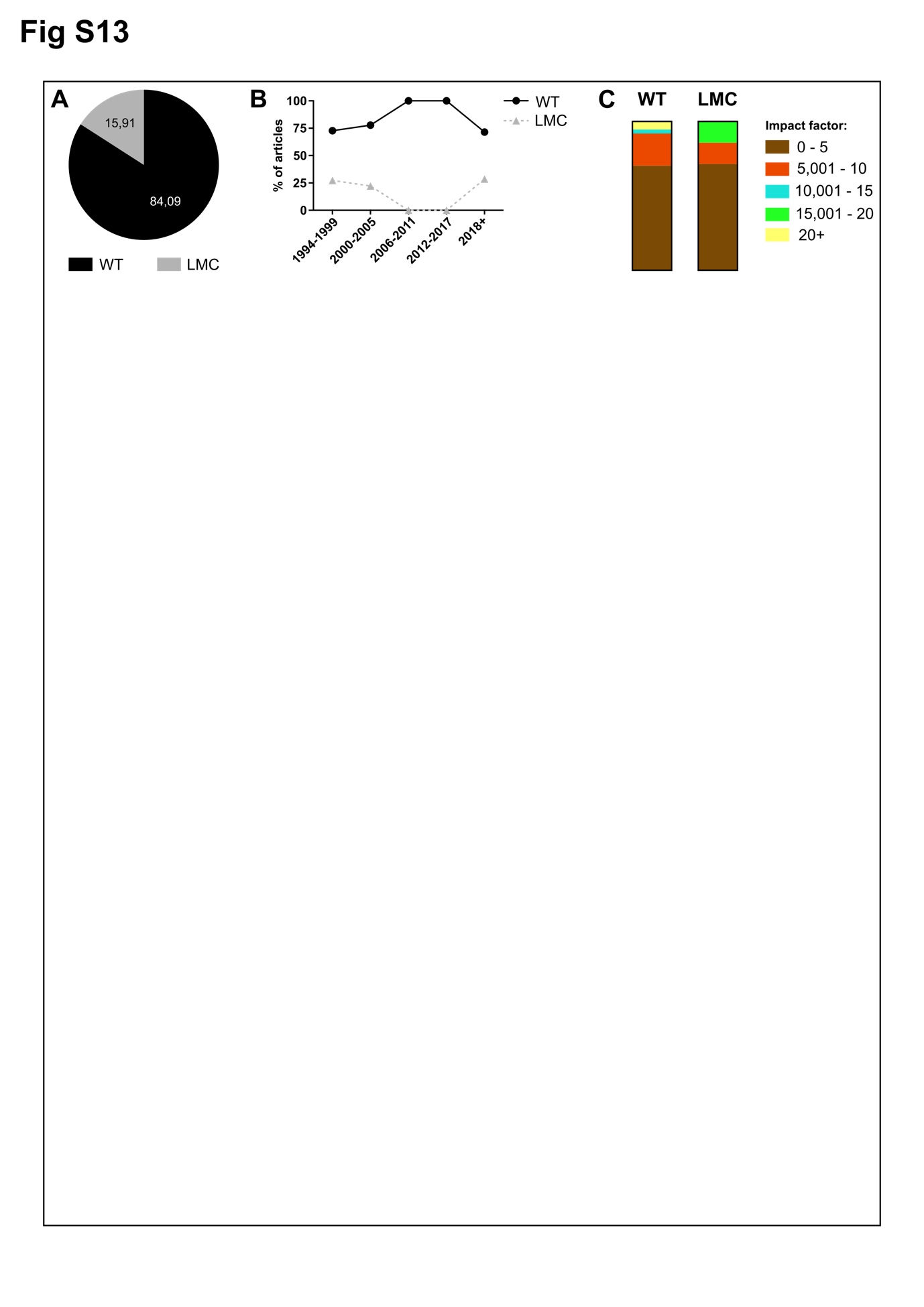

Supplement: Supplementary Figures [file EMS193947-supplement-Supplementary_Figures.docx]
